# The explosive radiation of *Cheirolophus* (Asteraceae, Cardueae) in Macaronesia

**DOI:** 10.1186/1471-2148-14-118

**Published:** 2014-06-02

**Authors:** Daniel Vitales, Teresa Garnatje, Jaume Pellicer, Joan Vallès, Arnoldo Santos-Guerra, Isabel Sanmartín

**Affiliations:** 1 Laboratori de Botànica – Unitat associada CSIC, Facultat de Farmàcia, Universitat de Barcelona, 08028 Barcelona, Catalonia, Spain; 2 Institut Botànic de Barcelona (IBB-CSIC-ICUB), 08038 Barcelona, Catalonia, Spain; 3 Jodrell Laboratory, Royal Botanic Gardens Kew, TW9 3DS Richmond, UK; 4 Jardín de Aclimatación de la Orotava, 38400 Tenerife, Spain; 5 Real Jardín Botánico (RJB-CSIC), 28014 Madrid, Spain

**Keywords:** Allopatric speciation, Canary Islands, Diversification, Island radiation, Mediterranean Basin, Phylogeography

## Abstract

**Background:**

Considered a biodiversity hotspot, the Canary Islands have been the key subjects of numerous evolutionary studies concerning a large variety of organisms. The genus *Cheirolophus* (Asteraceae) represents one of the largest plant radiations in the Canarian archipelago. In contrast, only a few species occur in the Mediterranean region, the putative ancestral area of the genus. Here, our main aim was to reconstruct the phylogenetic and biogeographic history of *Cheirolophus* with special focus on explaining the origin of the large Canarian radiation.

**Results:**

We found significant incongruence in phylogenetic relationships between nuclear and plastid markers. Each dataset provided resolution at different levels in *Cheirolophus*: the nuclear markers resolved the backbone of the phylogeny while the plastid data provided better resolution within the Canarian clade. The origin of *Cheirolophus* was dated in the Mid-Late Miocene, followed by rapid diversification into the three main Mediterranean lineages and the Macaronesian clade. A decrease in diversification rates was inferred at the end of the Miocene, with a new increase in the Late Pliocene concurrent with the onset of the Mediterranean climate. Diversification within the Macaronesian clade started in the Early-Mid Pleistocene, with unusually high speciation rates giving rise to the extant insular diversity.

**Conclusions:**

Climate-driven diversification likely explains the early evolutionary history of *Cheirolophus* in the Mediterranean region. It appears that the exceptionally high diversification rate in the Canarian clade was mainly driven by allopatric speciation (including intra- and interisland diversification). Several intrinsic (e.g. breeding system, polyploid origin, seed dispersal syndrome) and extrinsic (e.g. fragmented landscape, isolated habitats, climatic and geological changes) factors probably contributed to the progressive differentiation of populations resulting in numerous microendemisms. Finally, hybridization events and emerging ecological adaptation may have also reinforced the diversification process.

## Background

In recent decades, the Macaronesian archipelagos of Azores, Cape Verde, Madeira, Savages and Canary Islands have been the subject of numerous studies concerning patterns of colonization and speciation of different plant lineages [1-4]. In particular, the Canary Islands have drawn special attention from biogeographers because of their high degree of endemism, wide geological age ranges, variety of ecological conditions and unusual short distance to the mainland [5]. This has made the archipelago an ideal natural laboratory to test general hypotheses on island biogeography and evolution [5-9]. Recently, phylogenetic studies in Macaronesian plants have started incorporating information on lineage divergence times [3,10,11], a key factor when addressing evolutionary questions on the processes underlying lineage diversification [12] and their role in community assembly [13].

With approximately 20 endemic species, the genus *Cheirolophus* Cass. (1817) (Asteraceae, Cardueae) is considered one of the ten largest plant radiations in the Canary Islands [6]. In fact, ongoing taxonomical investigation points towards the existence of an even larger number of species (A. Santos-Guerra, unpubl. data). With the exception of *Ch. teydis* (C.Sm.) G.López from La Palma and Tenerife, all species are endemic to one of the central or western islands (Gran Canaria, Tenerife, La Gomera, La Palma and El Hierro). Most species present very narrow geographical ranges, but exhibit notable differences in their ecological preferences and morphological characteristics. Canarian *Cheirolophus* typically occur as small populations isolated on humid basalt cliffs. However, some species are adapted to live in remarkably different habitats, such as xeric environments (e.g. *Ch. junonianus* (Svent.) Holub), the subalpine zone (e.g. *Ch. teydis*) or coastal environments (*Ch. webbianus* (Sch.Bip.) Holub) [14]. This ecological diversity, coupled with a large species richness distributed in a clearly geographical pattern, makes *Cheirolophus* an ideal group to explore patterns and processes behind island diversification.

**Figure 1:**
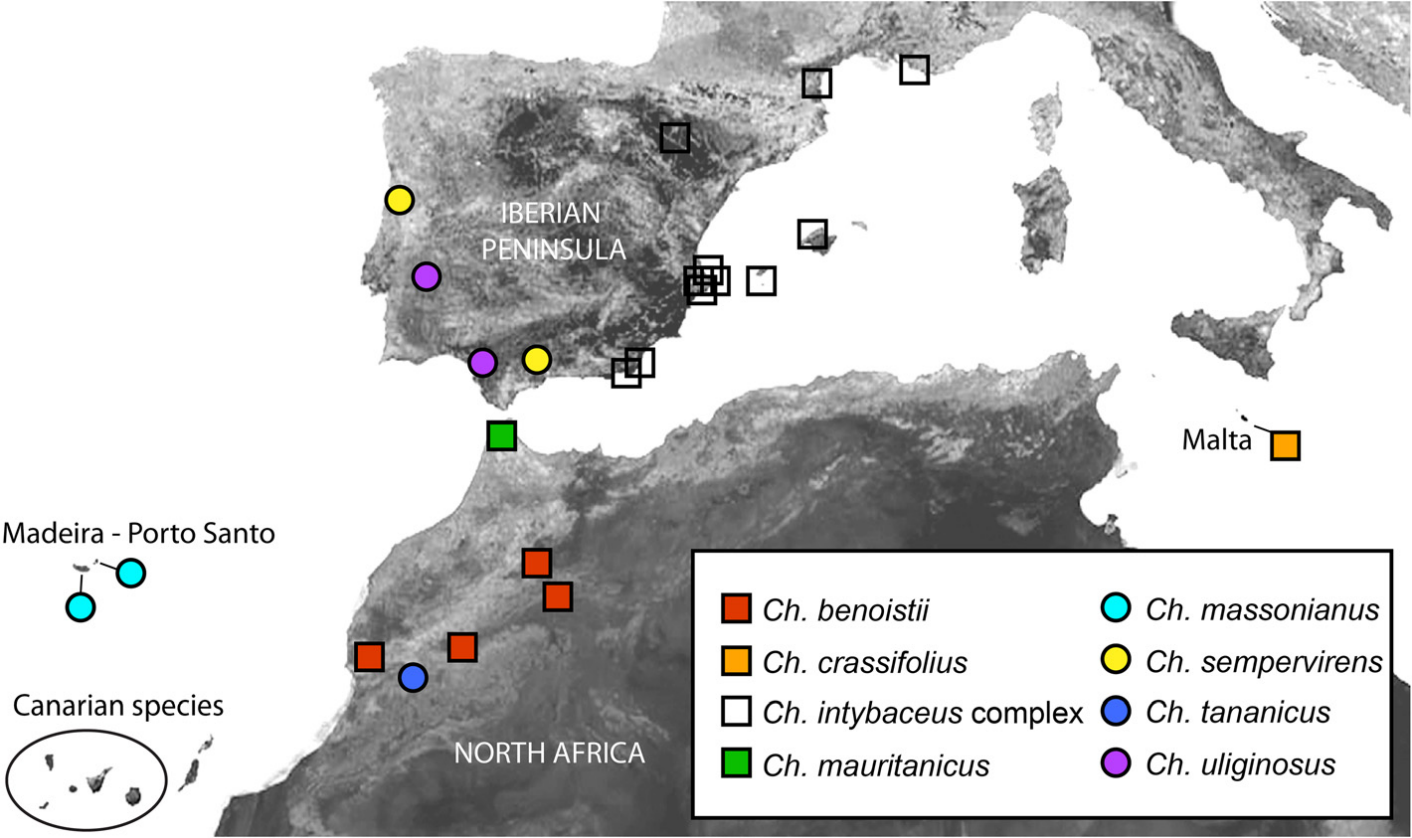
**Map with sampled localities.** Geographical distribution of the populations of *Cheirolophus* species included in this study. Further details on Canarian populations are given in Figure 4.

In addition to the large Canarian radiation, the genus occurs in Madeira [*Ch. massonianus* (Lowe) A.Hansen & Sunding] and the Western Mediterranean Basin, including the Mediterranean climate Atlantic coasts of the Iberian Peninsula (Figure 1). The species with the widest geographical distribution are the Mediterranean *Cheirolophus intybaceus* (Lam.) Dostál and the two Atlantic *Ch. sempervirens* (L.) Pomel and *Ch. uliginosus* (Brot.) Dostál [15]. There is considerable intraspecific variability within these species, especially in the *Ch. intybaceus* complex, which groups a set of morphologically similar taxa (e.g., *Ch. mansanetianus* Stübing, J.B.Peris, Olivares & J.Martín, *Ch. lagunae* Olivares & al.*, Ch. grandifolius* (Font Quer) Stübing & al., *Ch. intybaceus* var. *microcephala* Rouy). This has led to an unstable taxonomy, with no clear estimate of the number of species in the genus, which ranges from 25 to 30 depending on the author. All *Cheirolophus* species are perennial plants characterized by a thickened capitulum peduncle, a cypsela with deciduous pappus, *Serratula* pollen type [16], and a shrubby habit (except *Ch. uliginosus*, which is a perennial hemicryptophyte). They usually present an outcrossing mating system, although certain degree of self-compatibility has been reported [17]. From the conservation viewpoint, 22 species and subspecies of *Cheirolophus* are officially listed as vulnerable, endangered or critically endangered taxa, of which 17 are Macaronesian [18,19].

Earlier attempts to address phylogenetic relationships among *Cheirolophus* species or between the genus and its closest relatives have been based on allozymes [20] or DNA sequences from the nuclear ribosomal ITS and ETS regions [21,22]. These studies supported the existence of two well-defined major lineages: a Macaronesian clade, including all Macaronesian endemics, and a Mediterranean clade, grouping the North African *Ch. benoistii* (Humbert) Holub and *Ch. tananicus* (Maire) Holub with the *Ch. intybaceus* complex, distributed along the eastern shores of the Iberian Peninsula and southern France [22]. In contrast, lack of phylogenetic resolution within the Macaronesian clade - probably due to a recent history of colonization and diversification - prevented an in-depth study of phylogenetic relationships among the Macaronesian endemics [22]. More recently, a tribal phylogenetic reconstruction based on both nuclear and chloroplast markers [23] placed *Cheirolophus* in a basally branching position within the subtribe Centaureinae, as sister-group to the *Myopordon*-*Rhaponticum* lineage. Using new fossil evidence for Asteraceae, these authors estimated the divergence of *Cheirolophus* from its sister genera around the Early Miocene [23].

Here, we used nuclear and chloroplast DNA sequence data and the most comprehensive sampling of the genus conducted so far - including the entire species and infraspecific diversity registered in the International Plant Name Index - in conjunction with Bayesian phylogenetic analysis, divergence time estimation, macroevolutionary modelling, and biogeographical reconstruction to: (1) disentangle phylogenetic relationships within *Cheirolophus*, with special focus on the Canarian radiation, (2) infer the tempo and mode of lineage diversification within the genus, and (3) reconstruct the origin and colonization events in Canarian *Cheirolophus* in order to understand the factors underlying its large species richness.

## Methods

### Taxon sampling and DNA sequencing

DNA sequences were obtained from 57 populations representing 32 different taxa of *Cheirolophus* (Additional file 1: Table S1). *Serratula coronata* L., *Rhaponticoides hajastana* (Tzvelev) M.V.Agab. & Greuter, and *Rhaponticum pulchrum* Fisch. & C.A.Meyer were chosen as outgroup taxa based on previous phylogenetic studies of tribe Cardueae [20,22,23]. Total genomic DNA was extracted from silica gel-dried leaves and from herbarium specimens (ca. 10 mg) following the CTAB-protocol of Doyle and Doyle [24] with the modifications of Soltis et al. [25] and Cullins [26]. Nuclear rDNA regions (ITS and ETS) were newly sequenced for 11 taxa of *Cheirolophus* (eight species, three infraspecific taxa). This represents a 64% increase in the number of *Cheirolophus* species sequenced compared to previous studies [22]. For chloroplast DNA markers, a preliminary screening test involving 14 rapidly evolving cpDNA regions [27] was conducted (*ndhF-rpl32; prbA-trnH; psbD-trnT; psbE-petT; rpl32-trnL; rps16-trnK; trnD-rpoB; trnK-matK; trnL-trnF; trnQ-*5′*rps16; trnS-trnC; trnS-trnfM; trnT-trnG;* and *trnV-ndhC*). The regions that yielded the highest level of polymorphism were selected for further sequencing (*rpl32-trnL, rpoB-trnD, rps16-trnK* and *trnS-trnC*). All cpDNA sequences were newly generated for this study. In addition, we conducted a pilot study for the Macaronesian taxa to evaluate the within-population level of genetic diversity in the cpDNA markers. We analysed three individuals per population (when available), ensuring that these represented the entire area occupied by the population. Macaronesian populations showed no genetic variability for any of the plastid markers, so only one individual per population was included in further analyses.

DNA amplification procedures were performed as outlined by Pellicer et al. [28]. Details on primers used and polymerase chain reaction (PCR) conditions are given in the Additional file 2: Table S2. Depending on the quality of the amplification, products were purified using the QIAquick PCR Purification Kit (Qiagen Inc., Valencia, CA, USA) or DNA Clean and Concentrator™-5 D4004 (Zymo Research, Orange, CA, USA) following the manufacturer’s protocol. Direct cycle sequencing of the purified DNA segments was performed using the BigDye Terminator Cycle Sequencing v3.1 (PE Biosystems, Foster City, CA, USA) following the protocol recommended by the manufacturer. Nucleotide sequencing was carried out at the Centres Científics i Tecnològics of the University of Barcelona on an ABI PRISM 3700 DNA analyzer (PE Biosystems, Foster City, CA, USA). Details on species authorities, geographical localities for samples, and GenBank accession numbers are given in the Additional file 1: Table S1 of the supporting information.

Sequences were edited with Chromas LITE v2.01 (Technelysium Pty, Tewantin, Australia), and aligned manually with BioEdit version 7.0.5.3 [29].

### Phylogenetic analysis

Bayesian inference, implemented in MrBayes 3.2 [30], was used to estimate phylogenetic relationships among species of *Cheirolophus* based on individual analyses of the concatenate ITS + ETS (nrDNA) dataset and the concatenate four-plastid marker (cpDNA) dataset (each with 60 sequences: 57 *Cheirolophus* samples plus three outgroup taxa). Before concatenating the different plastid and nuclear regions, we checked for conflict among them. Incongruence was assessed (i) with the ILD test implemented in PAUP v. 4.0b10 [31], using a P-value of 0.01, and 1000 replications with heuristic search and random addition of sequences and excluding uninformative characters and (ii) by looking for nodes that were strongly supported (PP ≥ 0.95) in the Bayesian 50% majority rule consensus tree of one region/dataset but were not present in the consensus tree of the other region/dataset. No incongruence was observed among the cpDNA regions and between the two nuclear (ITS/ETS) markers, so they were concatenated in two independent datasets (cpDNA and nrDNA), which were analyzed separately. The General Time Reversible model (GTR) was selected as the most appropriate nucleotide substitution model for the cpDNA dataset, and the same model with among-site rate variation (GTR + G) for the nrDNA dataset based on the Akaike information criterion implemented in jModelTest 0.1 [32]. We did not partition the plastid and nuclear datasets by gene region in the Bayesian analyses because of the observed low genetic variation among sequences and to avoid over-parameterization. Gaps inferred during the alignment of the nrDNA and cpDNA regions were manually coded and modelled as different, binary partitions, using the F81-like restriction site model in MrBayes [33]. Two independent Markov chain Monte Carlo (MCMC) analyses with four Metropolis-coupled chains each were run for 5,000,000, sampling every 100 generation. The first 5,000 trees were discarded as the ‘burn-in’ period, after confirming that the average standard deviation of the split frequencies was < 0.01, and the potential scale reduction factor approached 1.0 for all parameters. The remaining samples were pooled to construct a majority rule consensus tree that approximates the posterior distribution of the phylogeny – visualized in FigTree 1.3.1 [34] – and to obtain clade posterior probabilities.

Both the ILD test and the node-comparison approach revealed the existence of significant incongruence between the cpDNA and nrDNA genomes, so we decided not to concatenate these two datasets in further analyses. The cpDNA tree was in general less resolved than the nrDNA tree and most cases of incongruence concerned poorly resolved relationships at the backbone of the tree, which may be explained by the low information content (variability) at the phylogenetic species level in this dataset in comparison with nuclear markers (see Additional file 3: Table S3). One exception was the incongruent position of the Madeiran endemic *Ch. massonianus*, which showed high support and significantly distant phylogenetic positions in both the cpDNA and nrDNA datasets (see Results below). Incongruence among gene trees can be attributed to different causes, being incomplete lineage sorting (ILS) and hybridization the most commonly reported in plant groups experiencing rapid radiations (e.g. [35-37]). To explore whether the incongruent position of *Ch. massonianus* could be the result of either reticulate evolution or ILS, we conducted an additional analysis under *BEAST [38]. This multilocus coalescent method is known to address ILS phenomena, whereas it is not able to resolve incongruence derived from hybridization. *BEAST uses a multispecies coalescent approach to estimate the most probable species tree given the unlinked multi-locus sequence data (i.e., the nrDNA and cpDNA datasets) and assumes no gene flow between after population/species divergence [37]. We constructed two partitioned, concatenate nuclear-plastid dataset: the first one including all *Cheirolophus* species and a second one excluding the taxon suspected of causing the major incongruence among gene trees (i.e. *Ch. massonianus*). Theoretically, a hybrid taxon included into a multi-locus phylogeny introduces homoplasy with clades that contain the hybrid parents, because hybrid taxa are supposed to be overall intermediate to the parental taxa since they contain a mosaic of parental characters [39]. Therefore, the removal of the hybrid taxon should increase the branch support for the clades that include the parental taxa –or their most closely related species– by decreasing the amount of homoplasy in the dataset [40]. For both analyses, the same model priors employed for the MrBayes phylogenetic analysis and the BEAST divergence time analysis (see below) were selected. The Markov chain was run for 5 × 10^7^ generations, sampling every 1000th generation. Tracer 1.4 [41] was used to check the convergence of the analyses and to confirm that the effective sample size (ESS) of each parameter are sufficiently large. Trees were summarized in a maximum clade credibility (MCC) tree obtained in TreeAnnotator 1.6.2 [42] and visualized in FigTree [34].

### Divergence time estimation and diversification analysis

We estimated species divergence times in *Cheirolophus* using a Bayesian-relaxed clock approach implemented in BEAST 1.7.1 [43]. The analysis was carried out only on the nrDNA dataset because of lack of variability and poor resolution in the cpDNA dataset at the species phylogenetic level (see above). Choice of model priors was based on the Path Sampling (PS) and Stepping Stone (SS) sampling methods in BEAST, which have been shown to outperform other marginal likelihood estimators in terms of consistency [44]. The birth-death model [45] was selected as the tree prior and the uncorrelated lognormal rate variation among branches as the clock prior, with a broad uniform distribution (10^−1^-10^−6^) for the mean rate and a default exponential prior for the standard deviation parameter. Speciation birth-death models can be problematic when multiple individuals per taxon/species are included in an analysis, since they assume that tips represent extant species completely sampled from the clade of interest [38]. Consequently, for the dating analysis, we included only one individual/sample per taxon (including subspecies), resulting in a 35-sequence data matrix. The GTR + G model was used as substitution model with a separate gap partition. The Markov chain was run for 5 × 10^7^ generations, sampling every 1000th generation. Tracer 1.4 [41] was used first to check the convergence and mixing of each parameter, and then to confirm that the effective sample size (ESS) of each parameter was sufficient to provide reasonable estimates of the variance in model parameters (i.e. ESS values > 200, after excluding a burn-in fraction of 10%). Trees were summarized in a maximum clade credibility (MCC) tree obtained in TreeAnnotator 1.6.2 [42] and visualized in FigTree [34]. Since there is no known fossil record of *Cheirolophus*, estimation of absolute lineage divergence times relied on secondary age constraints obtained from the molecular dated phylogeny of Barres et al. [23]. This study was based on five different fossil calibration points, including newly discovered fossils of Asteraceae [23], and constitutes the most complete dating analysis of tribe Cardueae to date. Barres et al. [23]’s estimate for the most recent common ancestor of *Serratula*, *Rhaponticum*, *Rhaponticoides*, and *Cheirolophus* was used to calibrate the root node in our phylogeny. To reflect the uncertainty in deriving age estimates from a more inclusive dated phylogeny, itself calibrated with the fossil record, we used a normal distribution prior [46] for the root node age parameter, with a median of 24.51 Ma and a standard deviation (SD) of 2.7 Ma to span the entire confidence interval (95% high probability density (HPD): 20.17–29.62 Ma) obtained by Barres et al. [23].

We used a diverse array of diversification statistics implemented in the programming language R (http://www.R-project.org, R Development Core Team 2012) to analyse the tempo and mode of species diversification in genus *Cheirolophus*. The package APE 2.7-3 [47] was used to construct a lineage-through-time (LTT) plot from the nrDNA BEAST chronogram, after pruning the outgroup taxa. We used the gamma statistic [48] implemented in the R package GEIGER [49] to test whether rates of diversification have been constant through time; since taxon sampling is complete in *Cheirolophus,* there was no need to use the MCCR test to correct this statistic [48]. The R package TreePar v.2.1 [50] was used to detect temporal changes in diversification rates. In particular, we make use of episodic birth-death models in which diversification rates are allowed to change at certain points in time (rate-shifts). Maximum likelihood optimization was used to simultaneously estimate diversification parameters – the net diversification rate (*r* = speciation minus extinction) and the extinction fraction (ϵ = the extinction to speciation ratio) – for each time interval together with the rate-shift times [50]. We used likelihood ratio tests (LRT) to compare nested models of increasing complexity with one, two, three, or four rate shifts (an arbitrary high value based on the size of our phylogeny), using a grid on shift times of 0.2 Myr steps.

TreePar can detect temporal changes in diversification rates, but does not allow the rate of diversification to vary among lineages. Instead, we used MEDUSA [51] implemented in GEIGER to locate the position of these rate shifts on the phylogeny. MEDUSA uses an AIC-based stepwise approach that compares the likelihood of piecewise models, in which r and ϵ are estimated at various points in the phylogeny. We also used the method-of-moments estimator [52], implemented in GEIGER, to estimate the rate of diversification in Canarian *Cheirolophus* under two extreme values of the extinction fraction (ϵ = 0, no extinction, and ϵ = 0.9 high rate of extinction). This method does not require a resolved time-calibrated phylogeny, and can thus be used to direct estimation of speciation rates in groups that underwent diversification recently. In order to obtain reliable confidence limits, we used the 95% HPD interval for the age of the crown node of the Canarian clade based on the BEAST analysis of the nrDNA dataset. Additionally, we estimated the probability of obtaining a clade with the same size and age as the Canarian clade given the global diversification rate inferred for the entire genus and at increasing extinction fractions (ϵ = 0, 0.5, 0.8, 0.9).

### Phylogeographic analysis

To infer the phylogeographical history of Canarian *Cheirolophus*, we constructed two additional datasets based on the original nrDNA and cpDNA matrices but subsampling only the Canarian taxa; in all 32 populations from 20 species were included in these further datasets. Two separate haplotype networks were constructed from each dataset to visually explore genetic diversity within each species using the software TCS v.1.21 [53]; insertions/deletions longer than one base pair were re-coded as single base pair mutations and these indels were treated as a fifth character state. The origin and timing of dispersal events in the colonization of the archipelago were inferred –independently for the nrDNA and for the cpDNA datasets – with the discrete-state continuous-time Markov chain (CTMC) model [54] implemented in BEAST 1.6.2 [42]. This composite CTMC phylogenetic-biogeographic model allows simultaneous estimation of phylogenetic relationships, lineage divergence times, ancestral ranges, and migration rates between geographic locations using Bayesian MCMC inference [55] and is similar to the Bayesian island biogeographic model described in Sanmartín et al. [8]. We used five geographical states corresponding to the islands where *Cheirolophus* is present: Gran Canaria, Tenerife, Gomera, La Palma, and El Hierro. Migration rates were modelled under both uninformative (mean = 1; SD = 0) and geographically informed priors, i.e., SD = 0 and mean equal to the normalized inverse distance between the centroids of two geographic locations [54]. To calibrate the phylogeographic analysis of Canarian populations based on cpDNA data, we carried out a new BEAST analysis using a dataset of 35 cpDNA sequences including one representative each of all *Cheirolophus* species, with identical settings as the BEAST nrDNA analysis above, except that substitution rates were modelled with GTR. The age of the crown-node of Canarian *Cheirolophus* estimated in this analysis was used to calibrate the root node height in the phylogeographic analysis of the 32-populations Canarian dataset (log normal distribution, mean = 1.22 Ma, SD = 0.4). The root node of the phylogeographic analysis based on the ITS + ETS data was calibrated with the age of crown Canarian *Cheirolophus* obtained in the BEAST analysis of the nrDNA 35-taxa dataset (log normal distribution, mean = 1.73 Ma, SD = 0.4). These phylogeographic analyses were run under a constant-size coalescent model and the uncorrelated lognormal molecular clock, based on the PS and SS selection, for 20 × 10^6^ generations, with all other settings identical to those used in the dating analyses. Finally, Bayesian Stochastic Search Variable Selection (BSSVS) was used to identify those rates (colonization routes) that were frequently invoked to explain the diffusion process [54]; these were saved as a KML file for visualization in Google Earth 6.2.2.6613. Given the small size of the phylogeny, we used a threshold value of Bayes Factors (BF) > 2 to consider a rate as well supported in the BSSVS analysis.

## Results

### Phylogenetic relationships and congruence among cpDNA loci

The main characteristics for all markers analysed are summarized in the Additional file 3: Table S3. The nrDNA dataset (ITS + ETS) for the ingroup taxa included 1138 aligned nucleotide positions, of which 140 (12.3%) were variable. The cpDNA dataset (*trnS*-*trnC* + *rpl32*-*trnL* + *trnD*-*rpoB* + *rps16*-*trnK*) included 3764 aligned positions, 58 (1.54%) of them variable. A total of 330 new sequences were generated and deposited in GenBank, of which 90 were from ITS + ETS and 240 from cpDNA markers (see Additional file 1: Table S1).

**Figure 2:**
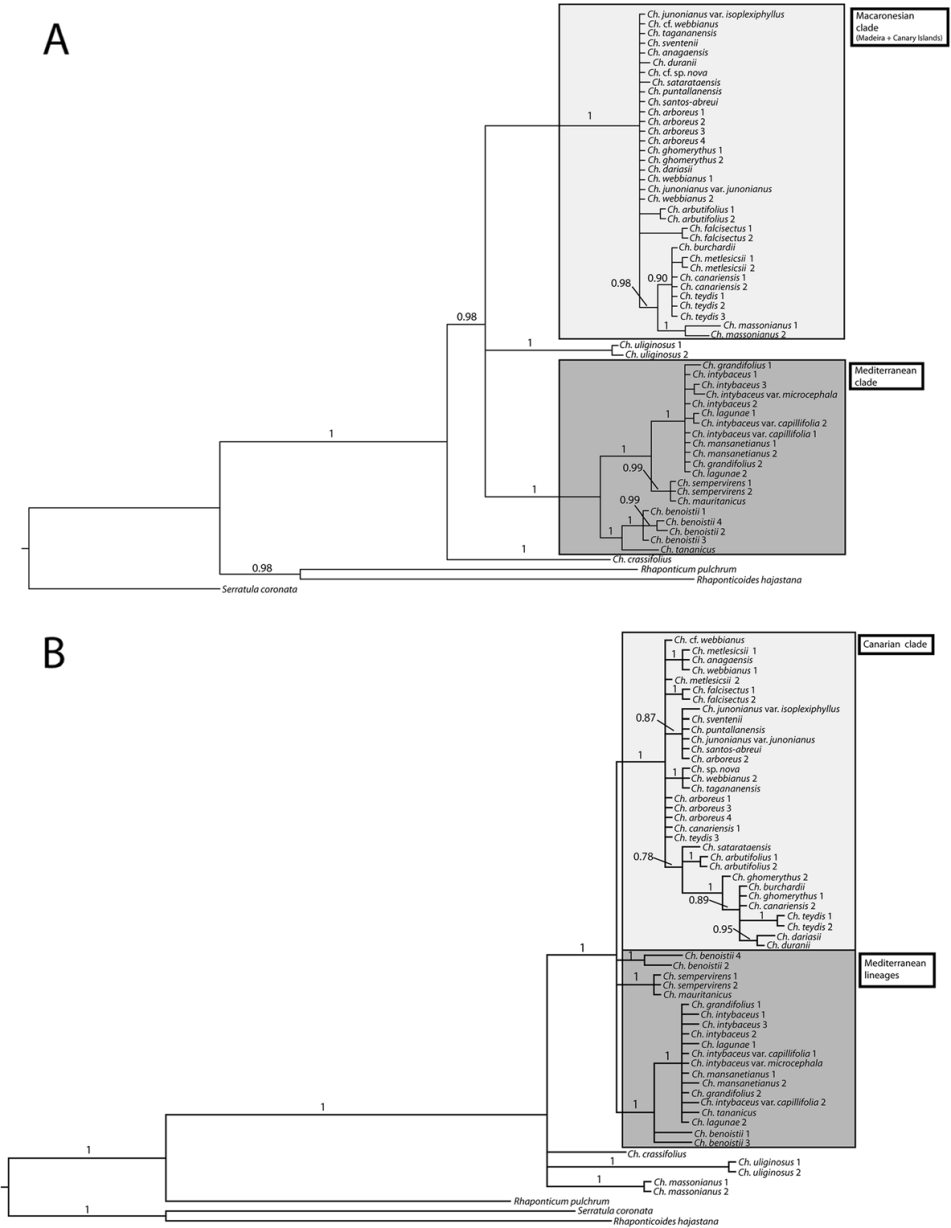
**Bayesian phylogenetic trees of *Cheirolophus *inferred from nuclear and plastid DNA datasets.** Majority-rule consensus tree resulting from a Bayesian analysis of **A)** the nuclear (ITS + ETS) dataset and the **B)** 4-marker chloroplast data set. Numbers above branches are Bayesian posterior probabilities (PP).

Figure 2 shows the consensus trees obtained from the separate Bayesian MCMC analysis of the nrDNA and cpDNA datasets. Phylogenetic relationships were generally better resolved in the nrDNA than in the cpDNA trees, especially for the backbone nodes. The opposite pattern was observed for the Canarian clade, which showed several well-supported subclades in the plastid phylogeny –even though some of these subclades are constituted by just one haplotype shared by several species (see below)– but poor resolution in the nrDNA tree. Both nuclear and plastid genomes support the monophyly of the genus (posterior probability, PP = 1.00) and recover a monophyletic “Macaronesian clade” (with or without *Ch. massonianus;* PP = 1.00, Figures 2A and B). In addition, the Mediterranean species appear grouped into three well-supported clades. These three clades form a well-supported “Mediterranean clade” in the nuclear dataset, but their relationships appear unresolved in the chloroplast phylogeny. *Cheirolophus crassifolius* (Bertol.) Susanna is recovered as the most basal lineage in the two trees (Figure 2). Significant incongruence between the nuclear and chloroplast phylogeny, as evidenced by node comparison and the ILD test (*p* < 0.001), concerned mainly conspecific samples grouped in the nuclear tree that appeared segregated into different clades in the plastid phylogeny, such as, the Mediterranean species *Ch. benoistii* or the Canarian *Ch. teydis* and *Ch. canariensis* (Brouss. ex Willd.) Holub (Figure 2). The only example of incongruence at the inter-species level concerned the position of the Madeiran endemic *Ch. massonianus*, which appears embedded within the Canarian clade in the nrDNA tree (Figure 2A), but occupies a position at the base of the genus together with *Ch. uliginosus* and *Ch. crassifolius* in the cpDNA phylogeny (Figure 2B). The multilocus coalescent *BEAST analysis including this species (see Additional file 4: Figure S1a) resulted in a tree topology in which the Madeiran taxon was placed at the base of the Canarian clade, in a position that was intermediate between the one it occupied in the cpDNA and the nrDNA trees (Figures 2A,B). In addition, the *BEAST analysis without *Ch. massonianus* (Additional file 4: Figure S1b) revealed increased clade support for those clades that include the putative parental taxa compared to the analysis including *Ch. massonianus* (i.e. the Canarian group, PP = 1.00 vs PP = 0.82; or *Ch. uliginosus*, PP = 0.55 vs PP = 0.47).

### Divergence time estimation and diversification analyses

**Figure 3:**
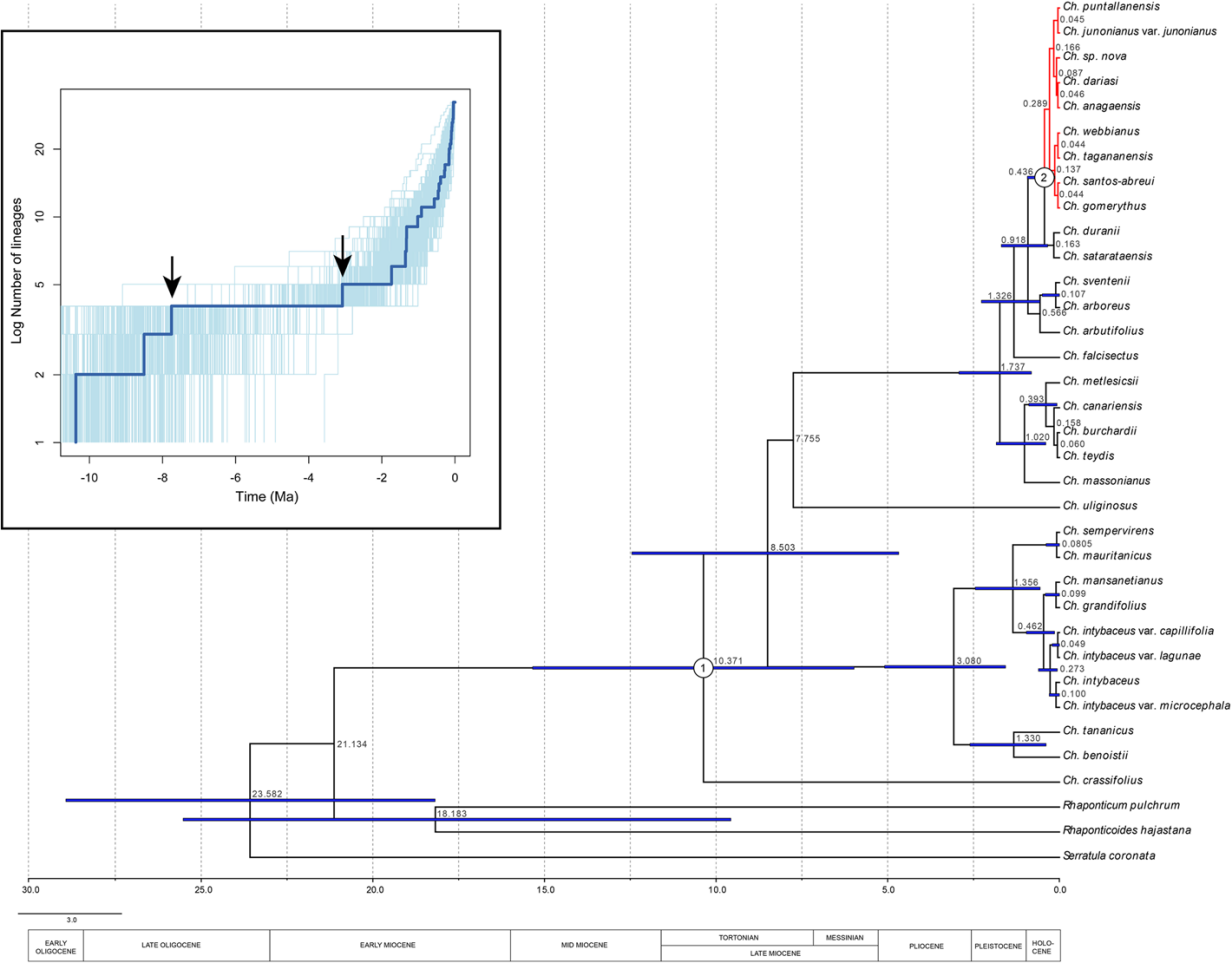
**Divergence time chronogram of *Cheirolophus *based on the nuclear DNA dataset.**BEAST maximum clade credibility tree, showing mean age estimates and 95% high posterior density intervals (for nodes with posterior probability > 0.5), obtained from the nuclear dataset. Significant changes in rates of diversification detected by MEDUSA are indicated at the appropriate node. The inset represents the lineage-through-time-plots (LTT) derived from the maximum clade credibility trees of the nuclear and chloroplast datasets. Faded lines represent the 95% confidence interval, as estimated from 1000 dated trees randomly sampled from the post burn-in BEAST distribution. Arrows indicate temporal shifts detected by TreePar.

Figure 3 shows the BEAST maximum clade credibility tree for the nrDNA dataset, whose topology is overall congruent and slightly better resolved than the one obtained from MrBayes, i.e., *Ch. uliginosus* is resolved as sister-group to the Macaronesian clade. Mean rates of evolution were estimated as 3.13 × 10^−9^ substitutions per site per year for nuclear regions, which is in agreement with average absolute rate of substitution for nrDNA estimated in other perennial angiosperms with relatively long generation times [56] like *Cheirophus* species. The mean age for crown-group *Cheirolophus* was 10.37 Ma (95% HPD confidence intervals: 5.98–15.35 Ma), while the divergence time between the Macaronesian clade from the Mediterranean lineages (Figure 3) was dated at 8.50 Ma (95% HPD: 4.68–12.45 Ma), and the crown-age of the Canarian clade at 1.74 Ma (0.82–2.93).

The LTT plot of the nrDNA chronogram showed an initial short phase of diversification (10–8 Ma) followed by a plateau between 8 and 3 Ma, with a final pronounced upturn in the rate of diversification c. 2 Ma (Figure 3, inset). This increase in diversification rates is supported by the gamma test, which rejects a constant-rate diversification model (5.730, *p* > 0.999). Although this test was designed to detect decreases in diversification rates compared to the constant-rate model [48], high positive values are usually interpreted as indicating an increase in the rate of speciation [12]. On the other hand, TreePar detected a decrease in diversification rates at 7.8 Ma, and an increase at 3.2 Ma for the model allowing two shifts (Figure 3). None of these rate shifts were, however, significant (LRT, *p* > 0.1), probably due to lack of statistical power when the phylogeny is small (n < 50 taxa). MEDUSA indicated a significant increase in diversification rates along the branch leading to a Canarian subclade (*r*_1_ = 0.00022; *r*_2_ = 6.55, c. 0.44 Ma, Figure 3)*.* The method-of-moments estimator indicated significantly higher diversification rates in the Canarian clade than expected, given their age and the global diversification rate for the entire genus (*r*_G_ = 0.2673; *p* < 0.05). This held under varying levels of the extinction fraction (ϵ = 0, 0.5, 0.8), except for a very high relative extinction rate of ϵ = 0.9 (*p* < 0.1), which is otherwise unrealistic for such a young group as estimated here for the Canarian clade. Diversification rates varied between r = 2.84–0.78 species Myr^−1^ for ϵ = 0) and 1.25–0.34 species Myr^−1^ for ϵ = 0.9.

### Phylogeography of Canarian *Cheirolophus*

**Figure 4:**
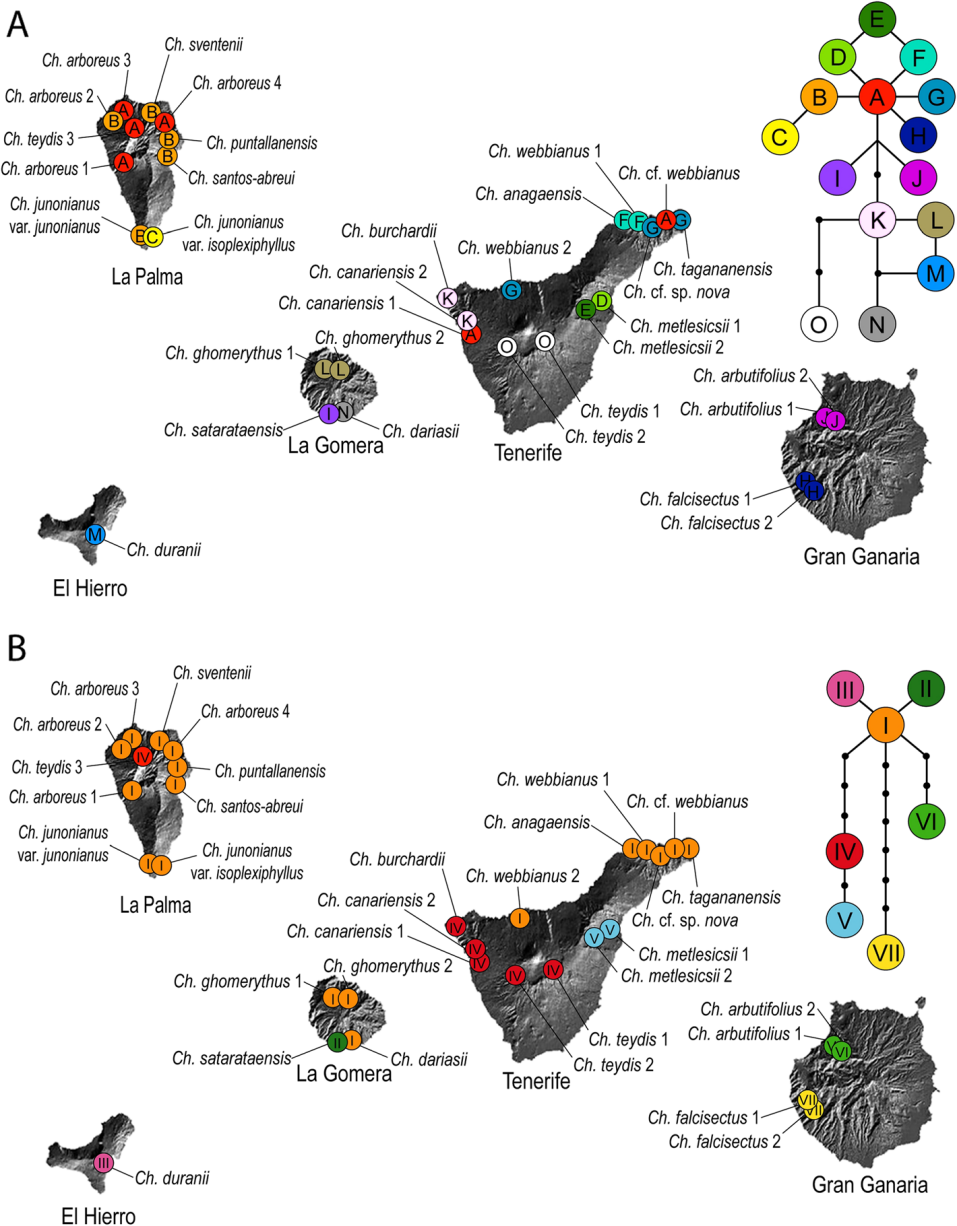
**Geographical distribution of populations and haplotypes of Canarian *Cheirolophus *sampled in this study*.*** The upper figure **(A)** shows plastid DNA haplotypes while the lower one **(B)** shows nuclear DNA haplotypes. The insets represent the haplotype networks estimated by TCS.

Plastid DNA regions showed higher variability within the Canarian clade than the nrDNA (ITS + ETS) regions. Twenty-one variable characters (see Additional file 3: Table S3) were found within the cpDNA regions, defining 15 haplotypes distributed among the sampled populations of Canarian *Cheirolophus* (Figure 4A). In contrast, nrDNA yielded 18 variable characters –most of them autapomorphic of one single species– that generated only seven different haplotypes (Additional file 3: Table S3; Figure 4b). Although population sampling was not dense enough to assess the entire genetic variability in each species, the cpDNA haplotype network (Figure 4A) indicated a clear geographical pattern, with all haplotypes confined to a single island, with the exception of haplotype A found in both Tenerife and La Palma. Haplotype B is the most widespread, occurring in five different species endemic to La Palma. No intra-population diversity was found, but several morphologically described species contained more than one haplotype distributed across the different sampled populations (Figure 4A). The species with the greatest haplotype diversity was *Ch. webbianus* (haplotypes A, F, and G), with a wide distributional range in Tenerife (Figure 4A). Tenerife is the island with the highest number of haplotypes (7), followed by La Palma and La Gomera (3 in each), Gran Canaria (2), and El Hierro (1). The haplotype network constructed from nrDNA data (Figure 4B) showed a less complex structure compared to the cpDNA network. Haplotype III is the most widely distributed, occurring in three different islands (Tenerife, La Gomera and La Palma) and eleven Canarian species. Haplotype IV is present also in several species (*Ch. canariensis*, *Ch. burchardii* Susanna and *Ch. teydis*) and in different islands (Tenerife and La Palma), whereas the rest of haplotypes are restricted to one single species. Contrary to the pattern observed in some taxa for the cpDNA data, all populations within a species showed the same nrDNA haplotype.

**Figure 5:**
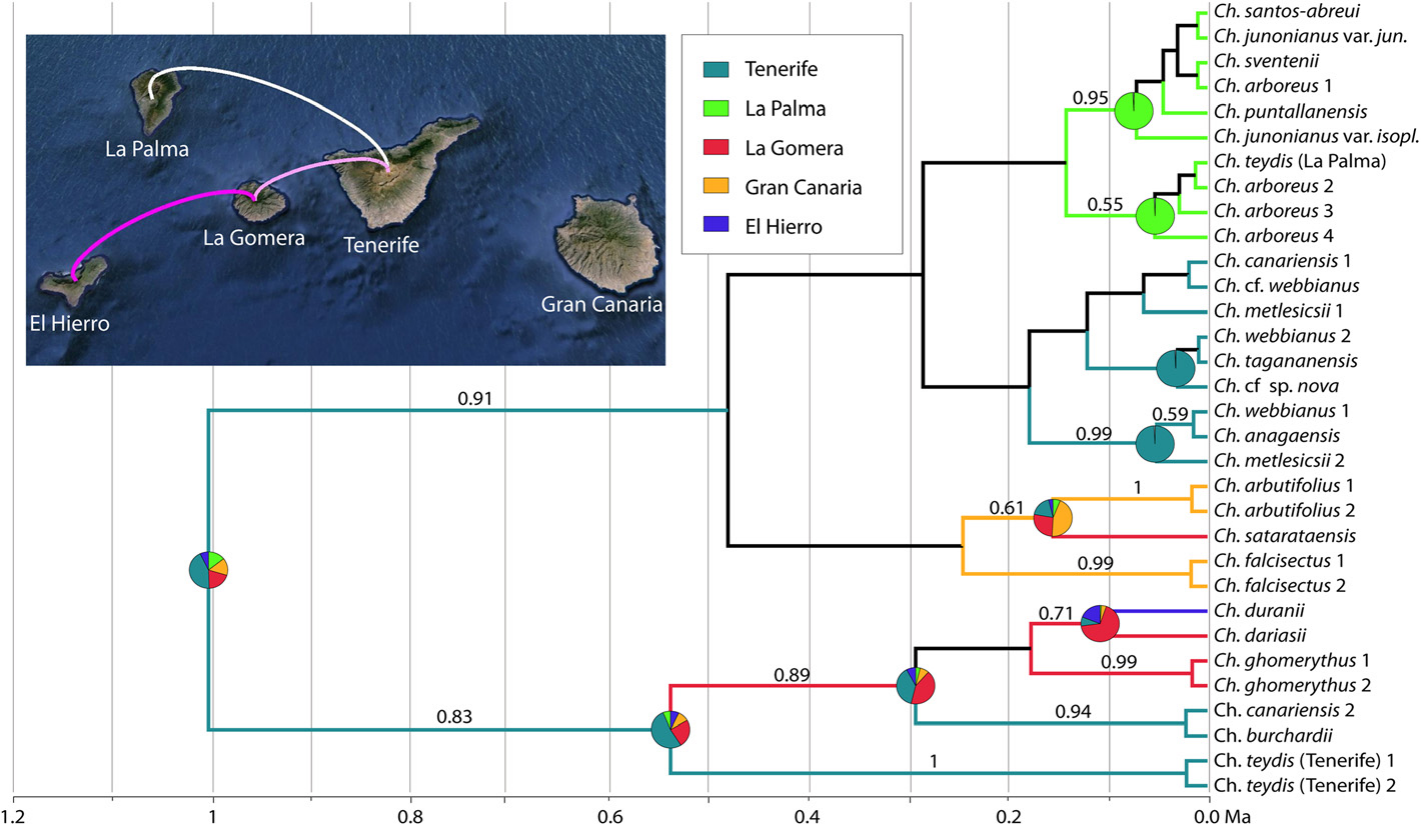
**Bayesian ancestral range reconstruction and colonization history of Canarian *Cheirolophus *based on plastid DNA markers.** Numbers above branches are Bayesian posterior probabilities (PP). The colored branch lengths represent the ancestral range with highest marginal probability for each lineage as inferred in BEAST (only branches with PP > 0.5). Node pie charts represent marginal probabilities for alternative ancestral ranges. Colonization routes identified by BSSVS are shown on the map with lines (see text for more details).

Figure 5 shows the ancestral ranges and the history of migration events across space and time in the Canarian lineage, as reconstructed in BEAST based on the plastid DNA dataset and using uninformative rate priors (geographically informed priors gave very similar results; data not shown). This analysis recovered several well supported clades within the Canarian lineage, even if some of them seem to be constituted by just one haplotype that is shared by several species (Figure 5). The first diversification event is dated around the Late Pleistocene: 1.02 Ma (0.42–1.96 Ma), with the majority of species diverging within the last 0.5 Ma (0.12–1.25 Ma). Some species were recovered as polyphyletic: *Ch. teydis*, *Ch. canariensis*, *Ch. webbianus*, *Ch. metlesicsii* Parada and *Ch. arboreus* (Webb & Berthel.) Holub. Tenerife is reconstructed as the most likely ancestral range of the Canarian radiation, from which several dispersal events took place eastward (from Tenerife to Gran Canaria) and westward (from Tenerife to La Gomera and La Palma, and from La Gomera to El Hierro). La Gomera was colonized twice, from Tenerife and from Gran Canaria, although the dispersal events concerning Gran Canaria are not recovered by BSSVS, probably due to low clade support and the small size of the phylogeny (Figure 5). Again, the phylogeographical analysis based on the nrDNA dataset was less informative than the one based on plastid markers: very few nodes received significant support while only one migration event (from Tenerife to La Palma) was recovered by the BSSVS analysis (Additional file 5: Figure S2).

## Discussion

### Hybridization and incongruence among plastid and nuclear genomes

The conflicting relationships found here between the nuclear and plastid phylogenies might be attributed to different coalescence-based and biological phenomena, including ILS, duplication/gene loss, chloroplast capture (introgression), polyploidy and hybridization. The nuclear ribosomal region (ITS + ETS) is by far the most widely used marker in plant systematics, and has been the preferred marker to disentangle phylogenetic relationships in Asteraceae (e.g., [52]), but phenomena like potentially non-functional pseudogene copy types and incomplete concerted evolution across the large multicopy tandem arrays in which the nrDNA is arranged, are known to cause problems in phylogenetic reconstruction, especially among closely related species [57,58]. Although this could be the case here, there is some evidence suggesting that the incongruence between nuclear and plastid genomes is more related to differences in genetic variability than to ILS in the nrDNA markers. On the one hand, we found no evidence of double bands in the PCR amplification, while polymorphic sites (double peaks in the electrophoretogram in which the weakest signal reached 25% of the strength of the strongest signal) represented less than 1% of sites in the ITS/ETS DNA sequences (these sites were not included in the phylogenetic and phylogeographic analysis). On the other, the topology and grouping of taxa in the nrDNA tree agrees well with the current species circumscription, reflecting geographical and morphological affinities, whereas some conspecific sequences fell into different clades in the cpDNA tree. Moreover, the nrDNA dataset contained at least ten-fold more variable sites than the full cpDNA dataset (see Additional file 3: Table S3), suggesting that the little variability in the latter (<2.0%) and associated homoplasy might be responsible for the artifactual positions of some species and lack of resolution at the base of the tree. Conversely, because of the their haploid nature, chloroplast markers generally require less time to fix novel mutations and present shorter coalescence times and higher polymorphism at lower taxonomical or population levels than nuclear ribosomal markers, despite their generally slower substitution rate [59]. Thus, they have been the marker of choice in species-diagnostic and phylogeographical studies on islands, outperforming the nuclear markers [60,61]. In our study, the cpDNA tree showed considerably higher levels of variability and phylogenetic resolution within the Canarian clade than the ITS + ETS tree, recovering several well-supported clades.

Finally, there is some evidence that at least part of the incongruence observed here can be caused by hybridization. Interspecific hybridization within groups that have recently radiated has been reported in many Macaronesian taxa [1,60,62,63] and might be behind the incongruent position of the Madeiran endemic *Ch. massonianus* in the nrDNA and cpDNA trees. This is supported by the position of this species in the multispecies coalescent analysis (*BEAST) intermediate between those occupied in the individual gene trees, and by the increased branch support for those clades including the putative parental taxa when this species was removed (Additional file 4: Figure S1), which are two evidences usually associated to horizontal gene transfer or hybridization (e.g. [39,40]). In addition, a genome size survey [22] revealed that the nuclear DNA content in the *Cheirolophus massonianus* (2C = 1.44 pg) was intermediate between those found in continental (mean 2C = 1.58 pg) and Canarian species (mean 2C = 1.38 pg). One potential explanation is hybridization or chloroplast capture (introgression) between a Canarian ancestor and the Atlantic Iberian endemic *Ch. uliginosus*, which occupies a basal position in the cpDNA tree together with *Ch. massonianus*. A close evolutionary relationship between species from Madeira and the western Iberian Peninsula has been documented in other studies [2], and is supported by the finding that submerged seamounts between Madeira and the continent might have acted as stepping stones during the Pleistocene glaciations [64]. Besides, preliminary amplified fragment length polymorphism (AFLP) analyses (Vitales et al., submitted) cluster *Ch. massonianus* within other Canarian *Cheirolophus* species, supporting the relationship depicted by the nrDNA tree.

Hybridization and introgression might also explain the polyphyletic nature of several Canarian species in the cpDNA tree (Figures 2 and 5). The only accession of *Ch. teydis* from La Palma exhibits haplotype A, which is very different from haplotype O of *Ch. teydis* populations occurring in Tenerife, but it is widely distributed over neighbouring populations of *Ch. arboreus*. Besides, the population of *Ch. arboreus* from north-western La Palma (Barranco Briestas) presents haplotype B, characteristic of other species from the island (Figure 4B); this latter population also exhibits slight morphological differences with respect to conspecific populations in the same island [19]. Furthermore, both *Ch. teydis* form La Palma and *Ch. arboreus* from Barranco Briestas show considerable levels of genetic admixture according to a preliminary AFLP analyses (Vitales et al.*,* submitted), supporting the hypothesis of ongoing gene flow. In Tenerife, discordant accessions of *Ch. canariensis* or *Ch. webbianus* presented haplotypes found in other geographically close species (*Ch. burchardii* and *Ch. anagaensis*, respectively) (Figure 4B). However, the relatively low sample size at the population level does not provide enough information to discern whether these latter cases of haplotype sharing are due to retention of ancestral polymorphism or actual gene flow among species, especially given the young age of the Canarian radiation (Figure 3). For example, the occurrence of haplotype A, ancestral according to its central position in the parsimony network (Figure 4), in accessions of three of the species recovered as polyphyletic (*Ch. teydis*, *Ch. canariensis*, and *Ch. webbianus*), might be explained by retention of ancestral polymorphisms due to insufficient time for coalescence. Further population-level studies are needed with intra-population sampling to discriminate among these explanations.

### Early evolutionary history of *Cheirolophus*

Given the different level of genetic variation and potential hybridization between markers mentioned above, the evolution of the genus is based here on the nrDNA tree, whereas the divergence and biogeographic history of the Canarian clade is discussed based mainly on evidence from the cpDNA population-level analysis, albeit considering the potential of hybridization.

In reconstructing the biogeographic history of tribe Cardueae, Barres et al. [23] placed the origin of the derived subtribe Centaureinae in West Asia, followed by repeated dispersal events across the Mediterranean region during the Miocene that gave rise to most extant genera. Our nrDNA phylogeny supports this scenario and dates the first diversification event in *Cheirolophus* during the Mid-Late Miocene (Figure 3). At that time, the Mediterranean Basin still featured tropical climate characteristics, but a progressive aridification starting in the east around 11–9 Ma [65] might have pushed *Cheirolophus* westward, explaining its current Western Mediterranean distribution. The basal position within the genus of *Ch. crassifolius*, endemic to Malta in the Central Mediterranean, agrees well with this hypothesis of an early east-to-west migration.

By the late Miocene, three additional extant lineages in the genus had diverged (Figure 3): the Western Mediterranean and Macaronesian clades, and the lineage formed by the single species *Ch. uliginosus*, a rare herbaceous member of the genus. This initial period of diversification was followed by a transition period of 5 Myr characterized by no apparent diversification ending in a sharp increase in the rate of diversification (Figure 3). Either a period of stasis followed by a recent radiation or a scenario of high extinction rates – constant or punctual – removing the early lineages, might explain the phylogenetic pattern found here (Figure 3; [12]). Although these two scenarios are difficult to distinguish on the basis of extant data alone [50], several lines of evidence support the high extinction hypothesis. TreePar detected a decrease in diversification rates at 7.8 Ma (Figure 3), and MEDUSA estimated high relative extinction rates in *Cheirolophus*, prior to the rate shift within the Canarian radiation (Figure 3). This slowdown in diversification could be explained by the effects of extinction associated with the extreme drought trend that culminated with the Messinian salinity crisis [65], which led to the replacement across the Mediterranean Basin of an ancestral “tropical-like” flora by new sclerophyllous plant communities [66]. Extant *Cheirolophus* lineages might have survived this hostile environment by migrating westwards, as exemplified by the Macaronesian clade or its putative sister-group, *Ch. uliginosus*, endemic to the humid Atlantic coast of the Iberian Peninsula. Others seem have developed ecological adaptations to drought environments, i.e. severe leaf reduction is observed in *Ch. benoistii* from the western Mediterranean clade and succulent leaves are present in *Ch. crassifolius* from Malta. A new increase in diversification rates was detected by TreePar at c. 3 Ma (Figure 3), coincident with the establishment of the Mediterranean-type climate around 3.5 Ma [66]. Although the start of diversification within the Mediterranean clade preceded that of the Macaronesian radiation (Figure 3), estimated divergence times for cladogenetic events leading to main subclades or species complexes were surprisingly synchronous. This synchronicity might be explained by the effect of Pleistocene climate oscillations [10], which played an important role in driving plant diversification in the Mediterranean region [67]. A similar pattern of diversification as the one described here, with a slowdown in diversification around 8–7 Ma and a subsequent increase at 3 Ma, has been observed in other Mediterranean plant taxa [68], supporting the hypothesis that Miocene climate changes governed the diversification of these lineages.

### Colonization and rapid diversification in the Canary Islands

A single colonization event to the Canary Islands was supported by both the nrDNA and cpDNA trees, in agreement with previous studies based on ITS alone and/or a more restricted sampling [21,22]. Following this initial colonization, *Cheirolophus* seems to have diversified rapidly: with c. 20 species arising in less than 1.8 million years (Figure 3). The high rate of diversification estimated for Macaronesian *Cheirolophus* (0.34–2.84 species Myr^−1^) is comparable to those exhibited by other island radiations. For example, Hawaiian *Bidens* (0.3–2.3 species Myr^−1^) and Macaronesian *Echium* (0.4–1.5 species Myr^−1^) were considered as the fastest plant radiations on volcanic islands to date [69]. Taking into account the area covered by both the Canary Islands and Madeira (8,321 km^2^), Macaronesian *Cheirolophus* may well represent the highest per-unit-area rate of diversification (4.09 × 10^−5^ to 3.41 × 10^−4^ species Myr^−1^ km^−2^) observed so far in plants [69-71]. One note of caution, however, must be added here concerning the use of species macroevolutionary models to estimate diversification rates; these models assume complete divergence between taxa [48,50,52,72], whereas in recently diverged groups such as *Cheirolophus* (see also the cases of *Lupinus* in the Andes [71] or *Tetramolopium* in Hawaiian Islands [73,74]) there might not have been enough time for complete sorting of alleles into the diverging lineages. Nevertheless, preliminary AFLP results indicate that all the described Macaronesian species form significantly distinct genotypic clusters (Vitales et al., submitted), thus supporting their taxonomic boundaries.

Which were the mechanisms underlying such rapid diversification? Geographical isolation and allopatric speciation undoubtedly played a significant role. A complex pattern of inter-island colonization events to the east and west was recovered in our phylogeographical analysis, which highlighted Tenerife as the main source area (Figure 5). This agrees with other Canarian studies (e.g., [7,10,60,75]), showing the central island as a major hub for colonization events. Indeed, Tenerife harbours the highest genetic diversity for *Cheirolophus* in the archipelago (Figure 4), a fact observed also in other Canarian genera such as *Bystropogon*[76], *Sideritis*[63] or *Aeonium*[1]. This higher diversity has traditionally been attributed to its ancient and complex palaeogeographic history. Tenerife is composed of three “palaeo-islands”, Anaga, Teno, and Roque del Conde, dating back between 4 and 12 Ma [77], which might have acted as a reservoir of relict biodiversity [5]. Most species of *Cheirolophus* in Tenerife are endemic to Teno (*Ch. canariensis*, *Ch. burchardii*) or Anaga (*Ch. anagaensis*, *Ch. tagananensis* (Svent.) Holub, *Ch.* cf. *sp. nova*), although their divergence largely postdates the origin of these ancient massifs. Another explanation is that habitat range fragmentation due to more recent events, such as the collapse of terrains during the last volcanic cycles (1.1–0.17 Ma, [78]) or the climatic fluctuations of the Pleistocene [79], might have contributed to the genetic isolation of populations (Figure 4). Finally, the patterns described here are constrained by the present distribution of the species and it is possible that they were different in the past. Intense volcanic activity and extinction might explain the currently low genetic diversity in older islands like Gran Canaria or the absence of *Cheirolophus* from the eastern islands of Fuerteventura and Lanzarote, which are closer to the mainland and are now too dry for *Cheirolophus* to grow.

Explosive intra-island diversification seems to have also occurred in La Palma, where most species originated after a colonization event from Tenerife less than 0.5 Ma (Figure 5). Limited seed dispersal and the rugged nature of the Canarian landscape have probably promoted rapid allopatric speciation events within the islands. Reduced dispersal potential in island organisms is known to be favoured by selection, as dispersal off the island is likely to result in the loss of the organism and/or propagules in the surrounding ocean [80,81]. Unlike most Centaureinae genera [82], the achenes in *Cheirolophus* can only be dispersed short distances by gravity (i.e. barochory). In addition, the sharp ravines and rocky cliffs which species of *Cheirolophus* inhabit provide deeply fragmented habitats that might have contributed to genetic isolation among populations, and subsequent allopatric speciation.

Nevertheless, several long distance dispersal (LDD) events (e.g. inter-island colonisations) occurred despite the apparently low ability of propagules to be transported. The ease of *Cheirolophus* for LDD is supported not only by the distribution of different species in five of the seven Canary Islands, but also by our haplotype network (Figure 4A) and phylogeographic reconstruction based on the plastid data (Figure 5), showing a double colonization of La Gomera (*Ch. ghomerythus* (Svent.) Holub - *Ch. dariasii* (Svent.) Bramwell and *Ch. satarataensis* (Svent.) Holub) and La Palma (endemic species and *Ch. teydis*). These double colonization events are also suggested by the less informative nrDNA haplotype network (Figure 4B). Given that stochasticity and non-standard transport mechanisms govern LDD in plants, drastic deviations from the usual dispersal distances do sporadically occur [83]. Indeed, evidence of transoceanic LDD has already been found for the Mediterranean *Ch. intybaceus*[84], suggesting that this phenomenon could be recurrent in the genus. Self-compatibility potential in *Cheirolophus*[17] may also have favoured the success of these colonization events, as dispersal of one single seed to a new habitat could establish a sexually reproducing population [85]. Finally, the basic chromosome number in *Cheirolophus* is *x* = 15, implying that the genus is originally polyploid [86,87]. This palaeopolyploidy could result in single fruits carrying higher genetic diversity – due to duplications – than what is expected in a diploid species, thus ameliorating the problem of severe genetic bottlenecks in the founding populations.

Although neutral genetic divergence as a result of restricted gene flow among isolated populations is probably the main force driving evolutionary diversification in Macaronesian *Cheirolophus*, ecological adaptation might be another mechanism responsible for this exceptionally rapid radiation. Even though it is not as spectacular as the case of the Macaronesian *Argyranthemum*[88] or the Hawaiian silversword alliance [89], some fine examples of intra-island ecological segregation can be found in *Cheirolophus*. For example, species such as *Ch. junonianus* from La Palma and *Ch. falcisectus* Svent. ex Montelongo & Moraleda from Gran Canaria, inhabiting xeric habitats, show clear leaf surface reduction, whereas their sister taxa (i.e. *Ch. arboreus* and *Ch. arbutifolius* (Svent.) G.Kunkel) occupying more humid locations in the same islands, display an arborescent habit and a larger leaf surface. Another example of ecological differentiation could be represented by *Ch. teydis*, the only *Cheirolophus* species inhabiting the subalpine zone (1800–2200 m) and showing morphological adaptations to tougher ecological conditions (i.e. rosette-like disposed leaves with reduced laminas; waxy leaf cover; high number of smaller flowers; annual flowering shoots). Given the short time since the start of the Canarian radiation (Figure 5), we are probably witnessing the initial stages of a process of phenotype-environment driven differentiation, although to demonstrate ecological differentiation and adaptive radiation more stringent tests than simple correlations are needed (see [90]).

## Conclusions

In the present study, we sequenced two nrDNA and four cpDNA regions from 57 populations representing the entire specific diversity in *Cheirolophus*. Significant incongruence was found in phylogenetic relationships between nuclear and plastid markers. The origin of *Cheirolophus* diversification was dated in the Mid-Late Miocene, followed by a slowdown in speciation rates at the end of the Miocene (Messinian) and a new increase in the Late Pliocene concurrent with the onset of the Mediterranean climate. Diversification within the Macaronesian clade started in the Early-Mid Pleistocene, with unusually high speciation rates (0.34–2.84 species Myr^−1^) giving rise to one of the most remarkable examples of explosive plant radiation in oceanic islands so far reported. This exceptionally high diversification rate was probably driven by allopatric speciation (both intra and inter-island diversification), favoured by several intrinsic (e.g. breeding system, polyploid origin, seed dispersal syndrome) and extrinsic factors (e.g. fragmented landscape, isolated habitats, climatic and geological changes) that contributed to the progressive differentiation of populations and resulted in numerous microendemisms. Finally, inter-specific genetic contact via hybridization and chloroplast capture events (see above) and emergent ecological adaptation could be other mechanisms reinforcing the diversification process in Macaronesian *Cheirolophus*.

### Data deposition

The data sets supporting the results of this article are available in the TreeBase repository, ID 15742 and http://purl.org/phylo/treebase/phylows/study/TB2:S15742[91].

## Competing interests

The authors declare that they have no competing interests.

## Authors’ contributions

AS, who has a deep knowledge on the Canarian flora, collected most of the plant material; DV, TG, JP, JV, and IS conceived and designed the experiments; DV performed the experiments; DV and IS analysed the data; DV and IS wrote the paper with relevant contributions of TG, JP and JV. All authors read and approved the final manuscript.

## Supplementary Material

Additional file 1: Table S1 Origin, collection data and GenBank accession numbers of the studied taxa. Click here for file

Additional file 2: Table S2 Regions used for DNA sequences, references and polymerase chain reaction (PCR) conditions. Click here for file

Additional file 3: Table S3 Characteristics of the aligned matrices for the nrDNA and cpDNA regions included in this study The values of the matrices containing all *Cheirolophus* species, only the Canarian *Cheirolophus* species and the *Cheirolophus* species plus the three outgroup species are given. Click here for file

Additional file 4: Figure S1 Multilocus coalescent analysis of *Cheirolophus* including **(a)** and excluding **(b)** the putative hybrid species *Ch. massonianus*. The analyses are based on the concatenated nrDNA and cpDNA datasets. Branch labels indicate posterior probability values. Click here for file

Additional file 5: Figure S2 Bayesian ancestral range reconstruction and colonization history of Canarian *Cheirolophus* based on nuclear DNA markers. Numbers above branches are Bayesian posterior probabilities (PP). The colored branch lengths represent the ancestral range with highest marginal probability for each lineage as inferred in BEAST (only branches with PP > 0.5). Node pie charts represent marginal probabilities for alternative ancestral ranges. Colonization routes identified by BSSVS are shown on the map with lines (see text for more details). Click here for file
