# Supplementary material for: The explosive radiation of Cheirolophus (Asteraceae, Cardueae) in Macaronesia
Source: BMC Evol Biol. 2014 Jun 2;14:118. doi: 10.1186/1471-2148-14-118 (PMC4048045; doi:10.1186/1471-2148-14-118)
Supplement: Additional file 1: Table S1 — Origin, collection data and GenBank accession numbers of the studied taxa. [file 1471-2148-14-118-S1.doc]

**Additional file 1. Origin, collection data and GenBank accession numbers of the studied taxa.**

| **Taxon** | **Locality/source** | **Voucher** | **GenBank accession numbers** | | | | | |
| --- | --- | --- | --- | --- | --- | --- | --- | --- |
|  |  |  | ***trnS–trnC*** | ***rpl32–trnL*** | ***rpoB–trnD*** | ***rps16–trnK*** | **ITS1, ITS2** | **ETS** |
| *Cheirolophus anagaensis* Santos | Tenerife: Anaga | Santos 14.V.08 (ORT) | KJ826305 | KJ826125 | KJ826185 | KJ826245 | KJ826035,  KJ826080 | KJ825990 |
| *Ch. arboreus* (Webb & Berthel.) Holub; Pop. 1 | La Palma: La Candelita | Acebedo 30.V.08 (ORT) | KJ826306 | KJ826126 | KJ826186 | KJ826246 | KJ826036,  KJ826081 | KJ825991 |
| *Ch. arboreus*; Pop.2 | La Palma: Barranco Briestas | Acebedo 31.V.08 (ORT) | KJ826307 | KJ826127 | KJ826187 | KJ826247 | KJ826037,  KJ826082 | KJ825992 |
| *Ch. arboreus*; Pop. 3 | La Palma: Barranco Fernando Porto | Acebedo 31.V.08 (ORT) | KJ826308 | KJ826128 | KJ826188 | KJ826248 | KJ826038,  KJ826083 | KJ825993 |
| *Ch. arboreus*; Pop. 4 | La Palma: Los Tilos | Acebedo 31.V 08 (ORT) | KJ826309 | KJ826129 | KJ826189 | KJ826249 | KJ826039,  KJ826084 | KJ825994 |
| *Ch. arbutifolius* (Svent.) G. Kunkel Pop. 1 | Gran Canaria: Agaete | Santos 17.II.09 (ORT) | KJ826310 | KJ826130 | KJ826190 | KJ826250 | KJ826040,  KJ826085 | KJ825995 |
| *Ch. arbutifolius* (Svent.) G. Kunkel Pop. 2 | Gran Canaria: Los Berrazales | Susanna 1420 (BC) | KJ826311 | KJ826131 | KJ826191 | KJ826251 | DQ131096, DQ131097 | DQ131075 |
| *Ch. benoistii* (Humbert) Holub; Pop. 1 | Morocco: Ksar es Souk | Susanna 1787 (BC) | KJ826312 | KJ826132 | KJ826192 | KJ826252 | AF045415, AF079945 | DQ131076 |
| *Ch. benoisitii*; Pop. 2 | Morocco: N of Er-Rachidia | Jury 17811 (K) | KJ826313 | KJ826133 | KJ826193 | KJ826253 | KJ826041,  KJ826086 | KJ825996 |
| *Ch. benoistii*; Pop. 3 | Morocco: Ait Said | Jury 17762 (K) | KJ826314 | KJ826134 | KJ826194 | KJ826254 | KJ826042,  KJ826087 | KJ825997 |
| *Ch. benoistii*; Pop. 4 | Morocco: Ouarzazate | Staundinger & Finckh (BC) 21.V.01 | KJ826315 | KJ826135 | KJ826195 | KJ826255 | KJ826043,  KJ826088 | KJ825998 |
| *Ch. burchardii* Susanna | Tenerife: between Buenavista and Teno | Susanna 1430 (BC) | KJ826316 | KJ826136 | KJ826196 | KJ826256 | AF021145, AF021162 | DQ131077 |
| *Ch. canariensis* (Brouss. ex Willd.) Holub; Pop. 1 | Tenerife: Barranco de Masca | Garnatje 2 (BC) | KJ826317 | KJ826137 | KJ826197 | KJ826257 | AF021151, AF021168 | DQ131078 |
| *Ch. canariensis*; Pop. 2 | Tenerife: Carrizales-Abache | Santos 17.VIII.08 (ORT) | KJ826318 | KJ826138 | KJ826198 | KJ826258 | KJ826044,  KJ826089 | KJ825999 |
| *Ch. crassifolius* (Bertol.) Susanna | Malta: pop. near La Valetta | Botanical Garden of la Valetta | KJ826319 | KJ826139 | KJ826199 | KJ826259 | AF021157, AF021174 | DQ131079 |
| *Ch. cf. webbianus* | Tenerife: Taganana, Roque de las Ánimas | Garnatje 1 (BC) | KJ826320 | KJ826140 | KJ826200 | KJ826260 | KJ826045,  KJ826090 | KJ826000 |
| *Ch. dariasii* | La Gomera: San Sebastian, El Cabrito | Mesa 01.V. 10 (ORT) | KJ826321 | KJ826141 | KJ826201 | KJ826261 | KJ826046,  KJ826091 | KJ826001 |
| *Ch. duranii* (Burchard) Holub | Spain. El Hierro: Temijiraque | Santos 23.VII.09 (ORT) | KJ826322 | KJ826142 | KJ826202 | KJ826262 | KJ826047,  KJ826092 | KJ826002 |
| *Ch. falcisectus* Montel. & Moral. Pop. 1 | Gran Canaria: San Nicolás de Tolentino | Susanna 1422 (BC) | KJ826323 | KJ826143 | KJ826203 | KJ826263 | AF021146, AF021163 | DQ131080 |
| *Ch. falcisectus* Montel. & Moral. Pop. 2 | Gran Canaria: Artéjevez | Santos 19.II.09 (ORT) | KJ826324 | KJ826144 | KJ826204 | KJ826264 | KJ826048,  KJ826093 | KJ826003 |
| *Ch. ghomerythus* (Svent.) Holub; Pop. 1 | La Gomera: La Tora | Mesa 08.V.10 (ORT) | KJ826325 | KJ826145 | KJ826205 | KJ826265 | KJ826049,  KJ826094 | KJ826004 |
| *Ch. ghomerythus*; Pop. 2 | La Gomera: Las Rosas | Santos 26.III.08 (ORT) | KJ826326 | KJ826146 | KJ826206 | KJ826266 | KJ826050,  KJ826095 | KJ826005 |
| *Ch. grandifolius* (Font Quer) Stübing, Peris, Olivares & Martín; Pop. 1 | Formentera: Sa Mola | Freixenet VIII.88 (BC) | KJ826327 | KJ826147 | KJ826207 | KJ826267 | DQ131098, DQ131099 | DQ131083 |
| *Ch. grandifolius*; Pop. 2 | Mallorca: Sa Talaia | Garnatje 222 (BC) | KJ826328 | KJ826148 | KJ826208 | KJ826268 | KJ826051,  KJ826096 | KJ826006 |
| *Ch. intybaceus* (Lam.) Dostál; Pop. 1 | Spain, Alacant: between Alcoi and Pego | Susanna 1249 (BC) | KJ826329 | KJ826149 | KJ826209 | KJ826269 | AF021152, AF021169 | DQ131082 |
| *Ch. intybaceus*; Pop. 2 | Spain, Zaragoza: Rueda de Jalón | Garnatje 225 (BC) | KJ826330 | KJ826150 | KJ826210 | KJ826270 | KJ826052,  KJ826097 | KJ826007 |
| *Ch. intybaceus*; Pop. 3 | France: Toulon | Garnatje 200 (BC) | KJ826331 | KJ826151 | KJ826211 | KJ826271 | KJ826053,  KJ826098 | KJ826008 |
| *Ch. intybaceus* (Lam.) Dostál. var. *capillifolius* Sandwith ex Lacaita; Pop. 1 | Spain, Alacant: Benitatxell, Cala Moraig | Garnatje 209 (BC) | KJ826332 | KJ826152 | KJ826212 | KJ826272 | KJ826054,  KJ826099 | KJ826009 |
| *Ch. intybaceus* var. *capillifolius*; Pop. 2 | Spain, Alacant: Benitatxell, Cim del Sol | Garnatje 208 (BC) | KJ826333 | KJ826153 | KJ826213 | KJ826273 | KJ826055,  KJ826100 | KJ826010 |
| *Ch. intybaceus* (Lam.) Dostál. var. *microcephala* Rouy | France, Languedoc-Roussillon: Leucate | Garnatje 197 (BC) | KJ826334 | KJ826154 | KJ826214 | KJ826274 | KJ826056,  KJ826101 | KJ826011 |
| *Ch. junonianus* (Svent.) Holub var. *junonianus* | La Palma: Fuencaliente, Roque Teneguía | Santos 26.VI.09 (ORT) | KJ826335 | KJ826155 | KJ826215 | KJ826275 | KJ826057,  KJ826102 | KJ826012 |
| *Ch. junonianus* (Svent.) Holub var. *isoplexiphyllus* (Svent.) G.Kunkel | La Palma: Fuencaliente, SW Volcan de San Antonio | Santos 20.I.08 (ORT) | KJ826336 | KJ826156 | KJ826216 | KJ826276 | KJ826058,  KJ826103 | KJ826013 |
| *Ch. lagunae* Olivares, Peris, Stübing & Martin; Pop. 1 | Spain, Alacant: Xàbia, Cap Prim | Vitatersana 442 (BC) | KJ826337 | KJ826157 | KJ826217 | KJ826277 | DQ 131100, DQ131101 | DQ131084 |
| *Ch. lagunae*; Pop. 2 | Spain, Alacant: Xàbia, La Caleta | Segarra 12.X.08 | KJ826338 | KJ826158 | KJ826218 | KJ826278 | KJ826059,  KJ826104 | KJ826014 |
| *Ch. mansanetianus* Stübing, Olivares, Peris & Martín;  Pop 1 | Spain, Almeria: Rodalquilar | Garnatje 214 (BC) | KJ826339 | KJ826159 | KJ826219 | KJ826279 | KJ826060,  KJ826105 | KJ826015 |
| *Ch. mansanetianus*; Pop. 2 | Spain, Almeria: barranco del Sabinar | Garnatje 215 (BC) | KJ826340 | KJ826160 | KJ826220 | KJ826280 | KJ826061,  KJ826106 | KJ826016 |
| *Ch. massonianus* (Lowe) A. Hansen & Sunding; Pop. 1 | Madeira: Cabo Girao | Barres 144 (BC) | KJ826341 | KJ826161 | KJ826221 | KJ826281 | KJ826062,  KJ826107 | KJ826017 |
| *Ch. massonianus ;* Pop. 2 | Porto Santo: Pico Branco | Barres 145 (BC) | KJ826342 | KJ826162 | KJ826222 | KJ826282 | KJ826063,  KJ826108 | KJ826018 |
| *Ch. mauritanicus* (Font Quer) Susanna | Morocco: Tétouan, mount Tissouka | Romo 4617 (BC) | KJ826343 | KJ826163 | KJ826223 | KJ826283 | AF021155, AF021172 | DQ131087 |
| *Ch. metlesicsii* Montel;  Pop. 1 | Tenerife: Arafo, Barranco Añavingo | Santos 01.VI.08 (ORT) | KJ826344 | KJ826164 | KJ826224 | KJ826284 | KJ826064,  KJ826109 | KJ826019 |
| *Ch. metlesicsii;* Pop. 2 | Tenerife: Arafo, Barranco Añavingo (Pop. 2) | Susanna 1427 (BC) | KJ826345 | KJ826165 | KJ826225 | KJ826285 | AF021150, AF021167 | DQ131088 |
| *Ch. puntallanensis* A. Santos | La Palma: Puntallana, Barranco Nogales | Santos 17.II.08 (ORT) | KJ826346 | KJ826166 | KJ826226 | KJ826286 | KJ826065,  KJ826110 | KJ826020 |
| *Ch. santos-abreui* A. Santos | La Palma: Barranco Madera | Santos 15.II.08 (ORT) | KJ826347 | KJ826167 | KJ826227 | KJ826287 | KJ826066,  KJ826111 | KJ826021 |
| *Ch. satarataensis* (Svent.) Holub | La Gomera: Alajeró | Santos 27.III.08 (ORT) | KJ826348 | KJ826168 | KJ826228 | KJ826288 | KJ826067,  KJ826112 | KJ826022 |
| *Ch. sempervirens* (L.) Pomel; Pop. 1 | Portugal: Coimbra, Antanhol | Vitales 12 (BC) | KJ826349 | KJ826169 | KJ826229 | KJ826289 | KJ826068,  KJ826113 | KJ826023 |
| Ch. *sempervirens*; Pop. 2 | Spain, Málaga: Ronda | Garnatje 274 (BC) | KJ826350 | KJ826170 | KJ826230 | KJ826290 | KJ826069,  KJ826114 | KJ826024 |
| *Ch.* cf. sp. *nova* A. Santos | Tenerife: Taganana, Afur | Santos 11.III.09 (ORT) | KJ826351 | KJ826171 | KJ826231 | KJ826291 | KJ826070,  KJ826115 | KJ826025 |
| *Ch. sventenii* (A. Santos) G. Kunkel | La Gomera: Barlovento | Santos III.08 (ORT) | KJ826352 | KJ826172 | KJ826232 | KJ826292 | KJ826071,  KJ826116 | KJ826026 |
| *Ch. tagananensis* (Svent.) Holub | Tenerife: Taganana, loc. cl. | Santos 30.VI.08 (ORT) | KJ826353 | KJ826173 | KJ826233 | KJ826293 | KJ826072,  KJ826117 | KJ826027 |
| *Ch. tananicus* (Maire) Holub | Morocco: Agadir, Tizi n’Test | Susanna 1395 (BC) | KJ826354 | KJ826174 | KJ826234 | KJ826294 | AF021153, AF021170 | DQ131091 |
| *Ch. teydis* (Chr. P. Sm.) G. López; Pop. 1 | Tenerife: El Teide, Boca de Tauce | Santos 11.V.08 (ORT) | KJ826355 | KJ826175 | KJ826235 | KJ826295 | KJ826073,  KJ826118 | KJ826028 |
| *Ch. teydis*; Pop. 2 | Tenerife: El Teide, Las Cañadas | Santos 09.IX.09 (ORT) | KJ826356 | KJ826176 | KJ826236 | KJ826296 | KJ826074,  KJ826119 | KJ826029 |
| *Ch. teydis*; Pop. 3 | La Palma: Garafía | Santos 12.VII.09 (ORT) | KJ826357 | KJ826177 | KJ826237 | KJ826297 | KJ826075,  KJ826120 | KJ826030 |
| *Ch. uliginosus* (Brot.) Dostál; Pop. 1 | Spain, Huelva: Mazagón, El Loro | Montserrat 1875 (BC) | KJ826358 | KJ826178 | KJ826238 | KJ826298 | AF021154, AF021171 | DQ131093 |
| *Ch. uliginosus*; Pop. 2 | Potugal, Portoalegre: Reguengo | Vitales 11 (BC) | KJ826359 | KJ826179 | KJ826239 | KJ826299 | KJ826076,  KJ826121 | KJ826031 |
| *Ch. webbianus* (Sch.Bip.) Holub; Pop. 1 | Tenerife: Anaga | Santos 14.V.08 (ORT) | KJ826360 | KJ826180 | KJ826240 | KJ826300 | KJ826077,  KJ826122 | KJ826032 |
| *Ch. webbianus*; Pop. 2 | Tenerife: Icod Beach, San Marcos | Santos 9.VII.09 (ORT) | KJ826361 | KJ826181 | KJ826241 | KJ826301 | KJ826078,  KJ826123 | KJ826033 |
| *Rhaponticoides hajastana* (Tzvelev) M. V. Agab. & Greuter | Armenia | Susanna 1587 et al. (BC) | KJ826362 | KJ826182 | KJ826242 | KJ826302 | AF319064, AF319118 | DQ131094 |
| *Rhaponticum pulchrum* Fisch. & C. A. Meyer | Russia | Popova 326 (LE) | KJ826363 | KJ826183 | KJ826243 | KJ826303 | KJ826079,  KJ826124 | KJ826034 |
| *Serratula coronata* L. | Botanical Garden of Vienna, Austria | (BC) | KJ826364 | KJ826184 | KJ826244 | KJ826304 | AY826327 | DQ310961 |
