# Supplementary material for: The explosive radiation of Cheirolophus (Asteraceae, Cardueae) in Macaronesia
Source: BMC Evol Biol. 2014 Jun 2;14:118. doi: 10.1186/1471-2148-14-118 (PMC4048045; doi:10.1186/1471-2148-14-118)
Supplement: Additional file 2: Table S2 — Regions used for DNA sequences, references and polymerase chain reaction (PCR) conditions. [file 1471-2148-14-118-S2.doc]

| Region | Primers | References | PCR conditions |
| --- | --- | --- | --- |
| ITS | 1406F | (White et al., 1990) | 94 ºC, 2 min; 30x (94 ºC, 1 min; 55 ºC, 30 s; 72 ºC, 3 m); 72 ºC, 15 m |
|  | ITS4 | (Nickrent et al. 1994) |  |
| ETS | ETS1f | (Linder et al 2000) | 95 ºC, 5 min; 30x (94 ºC, 45 s; 50 ºC, 45 s; 72 ºC, 40 s), 72 ºC, 7 min |
|  | 18S-2L | (Linder et al 2000) |  |
| *rpl32-trnL* | rpl32f | (Shaw et al., 2007) | 80ºC, 5 min; 30x (95ºC, 1 min; 50ºC, 1 min; 65ºC, 4 min); 65ºC, 5 min |
|  | trnL^UAG^ | (Shaw et al., 2007) |  |
| *trnS-trnC* | trnS^GCU^ | (Shaw et al., 2005) | 95ºC, 5 min; 30x (94ºC, 30 s; 62ºC, 1 min; 72ºC, 2 min); 72ºC, 5 min |
|  | trnC^GCA^R | (Shaw et al., 2005) |  |
| *rpoB-trnD* | rpoB | (Shaw et al., 2005) | 85ºC, 5 min; 35x (96ºC, 1 min; 52ºC, 2 min; 72ºC, 3 min); 72ºC, 5 min |
|  | trnD^GUC^R | (Shaw et al., 2005) |  |
| *rps16-trnK* | rpS16x2F2 | (Shaw et al., 2007) | 80ºC, 5 min; 35x (94ºC, 30 s; 50–55ºC, 30 s; 72ºC, 1 min); 72ºC, 5 min |
|  | trnK(UUU)x1 | (Shaw et al., 2007) |  |

**Additional file 2. Regions used for DNA sequences, references and polymerase chain reaction (PCR) conditions.**

White T.J., Bruns T., Lee S., & Taylor J. (1990) Amplification and direct sequencing of fungal ribosomal RNA genes for phylogenetics. *PCR Protocols: A Guide to Methods and Applications* (ed. by M. Innis, D. Gelfand, J. Sninsky, and T. White), pp. 315–322. Academic Press, San Diego.

Nickrent D.L., Schuette K.P., & Starr E.M. (1994) A molecular phylogeny of *Arceuthobium* (Viscaceae) based on nuclear ribosomal DNA internal transcriber spacer sequences. *American journal of botany*, **81**, 1149–1160.

Linder C.R., Goertzen L.R., Heuvel B. V, Francisco-Ortega J., & Jansen R.K. (2000) The complete external transcribed spacer of 18S-26S rDNA: Amplification and phylogenetic utility at low taxonomic levels in Asteraceae and closely allied families. *Molecular Phylogenetics and Evolution*, **14**, 285–303.

Shaw J., Lickey E.B., Beck J.T., Farmer S.B., Liu W., Miller J., Siripum K.C., Winder C.T., Schilling E.T., & Small R.L. (2005) The tortoise and the hare II: relative utility of 21 non coding chloroplast DNA sequences for phylogenetic analysis. *American Journal of Botany*, **92**, 142–166.

Shaw J., Lickey E.B., Schilling E.E., & Small R.L. (2007) Comparison of whole chloroplast genome sequences to choose noncoding regions for phylogenetic studies in angiosperms: the tortoise and the hare III. *American Journal of Botany*, **94**, 275–288.
