# Supplementary material for: The explosive radiation of Cheirolophus (Asteraceae, Cardueae) in Macaronesia
Source: BMC Evol Biol. 2014 Jun 2;14:118. doi: 10.1186/1471-2148-14-118 (PMC4048045; doi:10.1186/1471-2148-14-118)
Supplement: Additional file 3: Table S3 — Characteristics of the aligned matrices for the nrDNA and cpDNA regions included in this study The values of the matrices containing all Cheirolophus species, only the Canarian Cheirolophus species and the Cheirolophus species plus the three outgroup species are given. [file 1471-2148-14-118-S3.doc]

**Additional file 3. Characteristics of the aligned matrices for the nrDNA and cpDNA regions included in this study.** The values ​​of the matrices containing all *Cheirolophus* species, only the Canarian *Cheirolophus* species and the *Cheirolophus* species plus the three outgroup species are given.

| **Marker** | **ITS** | **ETS** | **nrDNA** | ***trn*S-*trn*C** | ***rpl*32-*trn*L** | ***rpo*B-*trn*D** | ***rps*16-*trn*K** | **cpDNA** |
| --- | --- | --- | --- | --- | --- | --- | --- | --- |
|  |  |  |  |  |  |  |  |  |
| **Length range (bp)** |  |  |  |  |  |  |  |  |
| *Cheirolophus* species | 468-471 | 638-653 | 1106-1124 | 808-875 | 941-953 | 993-1093 | 832-834 | 3574-3755 |
| Canarian species only | 469 | 638-639 | 1107-1108 | 807-809 | 944-946 | 1013-1073 | 833-834 | 3597-3662 |
| Outgroup included | 468-471 | 638-668 | 1106-1139 | 808-875 | 933-955 | 993-1241 | 832-862 | 3566-3933 |
| **Aligned length (bp)** |  |  |  |  |  |  |  |  |
| *Cheirolophus* species | 471 | 667 | 1138 | 875 | 955 | 1100 | 834 | 3764 |
| Canarian species only | 469 | 639 | 1108 | 853 | 946 | 1073 | 834 | 3706 |
| Outgroup included | 472 | 673 | 1145 | 879 | 969 | 1350 | 874 | 4072 |
| **Number of variable nucleotides** |  |  |  |  |  |  |  |  |
| *Cheirolophus* species | 69 | 71 | 140 | 5 | 25 | 15 | 13 | 58 |
| Canarian species only | 5 | 12 | 17 | 1 | 1 | 4 | 2 | 8 |
| **Number of indels** |  |  |  |  |  |  |  |  |
| *Cheirolophus* species | 3 | 3 | 6 | 3 | 6 | 8 | 2 | 19 |
| Canarian species only | 0 | 1 | 1 | 3 | 4 | 5 | 1 | 13 |
| **Total variable sites (nucleotide + indels)** |  |  |  |  |  |  |  |  |
| *Cheirolophus* species | 72 | 74 | 146 | 8 | 31 | 23 | 15 | 77 |
| Canarian species only | 5 | 13 | 18 | 4 | 5 | 9 | 3 | 21 |
