# Supplementary figures and images for: The explosive radiation of Cheirolophus (Asteraceae, Cardueae) in Macaronesia
Source: BMC Evol Biol. 2014 Jun 2;14:118. doi: 10.1186/1471-2148-14-118 (PMC4048045; doi:10.1186/1471-2148-14-118)

A

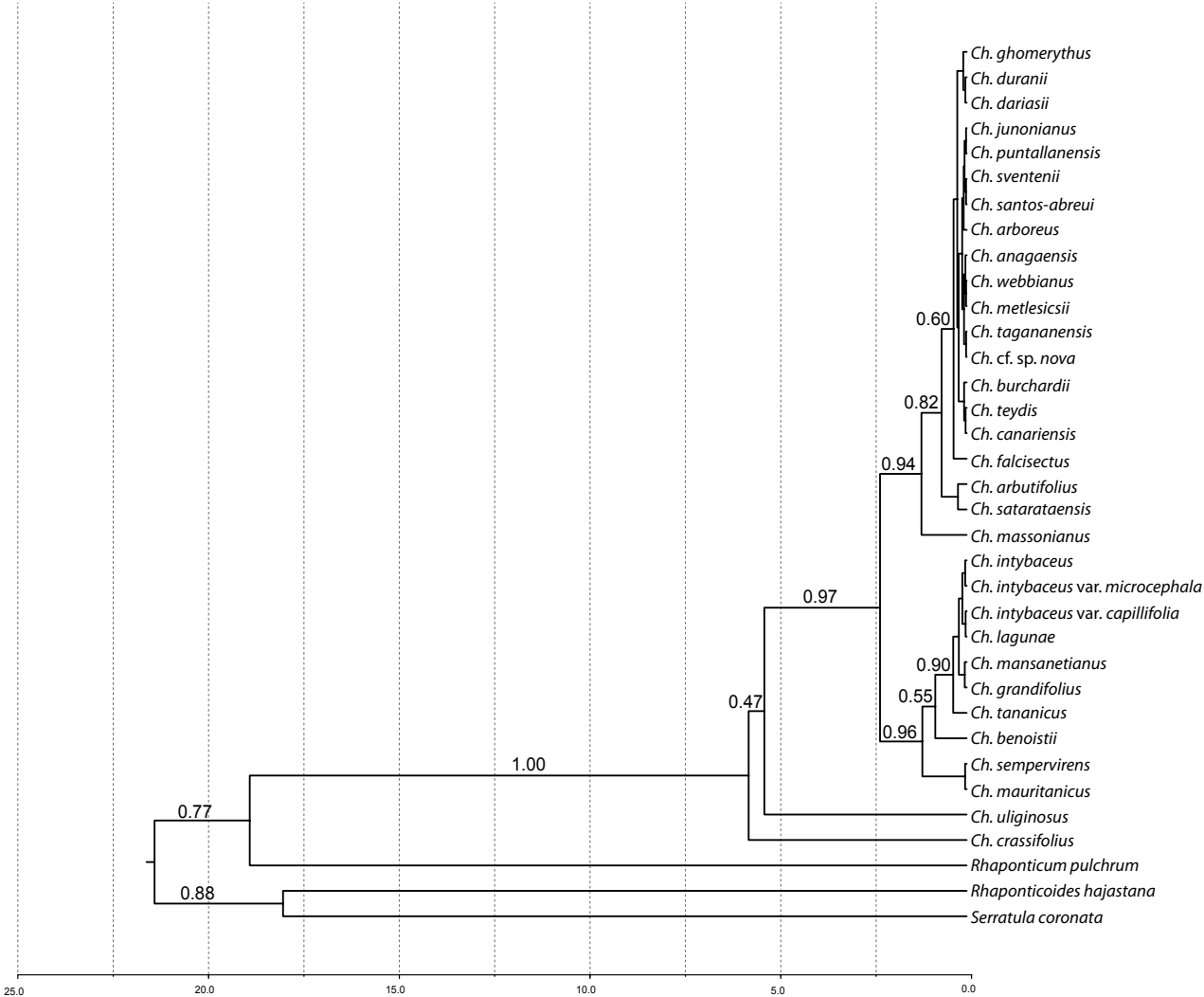

B

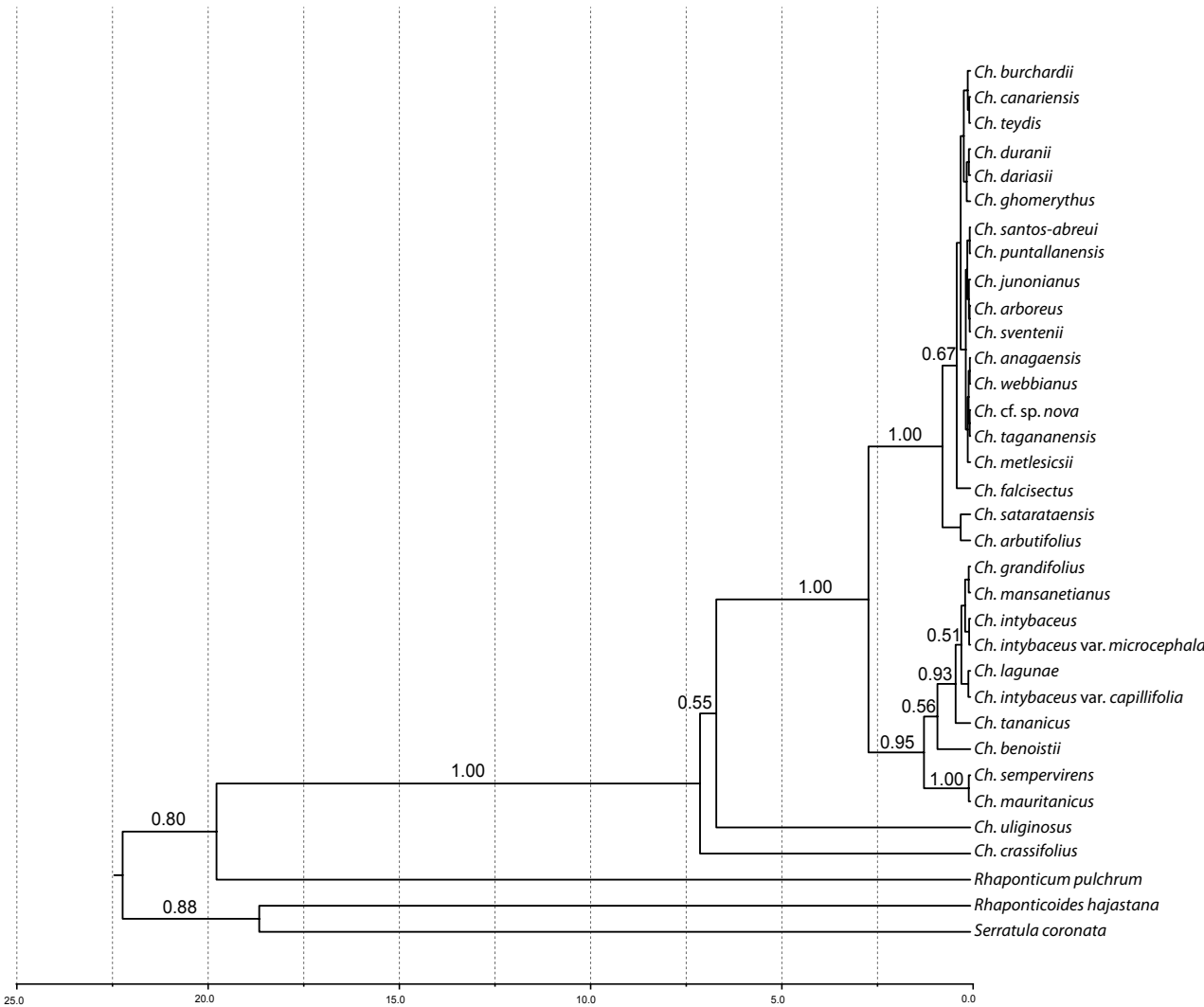

Supplement: Additional file 4: Figure S1 — Multilocus coalescent analysis of Cheirolophus including (a) and excluding (b) the putative hybrid species Ch. massonianus. The analyses are based on the concatenated nrDNA and cpDNA datasets. Branch labels indicate posterior probability values. [file 1471-2148-14-118-S4.pdf]

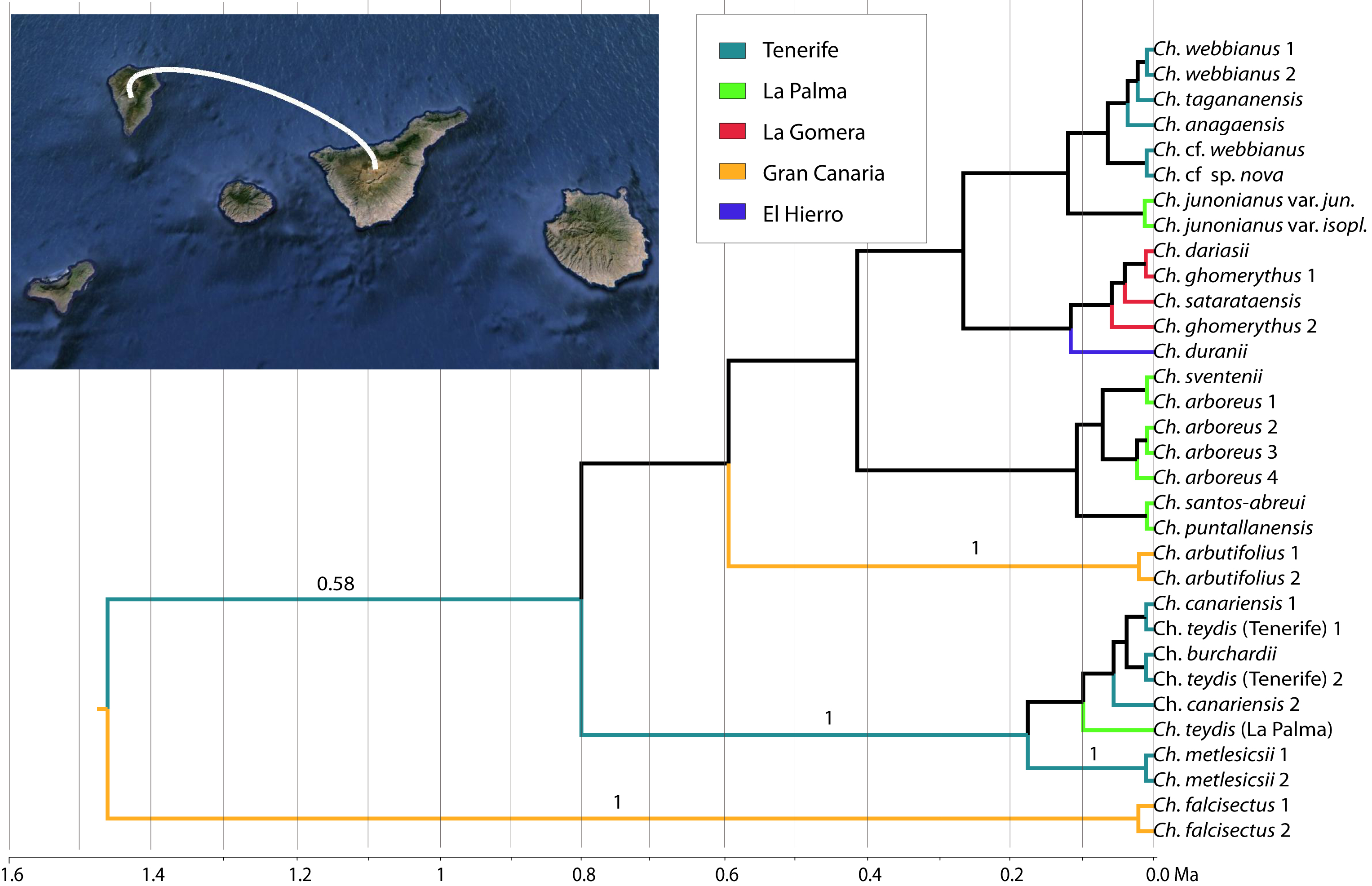

Supplement: Additional file 5: Figure S2 — Bayesian ancestral range reconstruction and colonization history of Canarian Cheirolophus based on nuclear DNA markers. Numbers above branches are Bayesian posterior probabilities (PP). The colored branch lengths represent the ancestral range with highest marginal probability for each lineage as inferred in BEAST (only branches with PP > 0.5). Node pie charts represent marginal probabilities for alternative ancestral ranges. Colonization routes identified by BSSVS are shown on the map with lines (see text for more details). [file 1471-2148-14-118-S5.jpeg]
